# One-step generation of complete gene knockout mice and monkeys by CRISPR/Cas9-mediated gene editing with multiple sgRNAs

**DOI:** 10.1038/cr.2017.81

**Published:** 2017-06-06

**Authors:** Erwei Zuo, Yi-Jun Cai, Kui Li, Yu Wei, Bang-An Wang, Yidi Sun, Zhen Liu, Jiwei Liu, Xinde Hu, Wei Wei, Xiaona Huo, Linyu Shi, Cheng Tang, Dan Liang, Yan Wang, Yan-Hong Nie, Chen-Chen Zhang, Xuan Yao, Xing Wang, Changyang Zhou, Wenqin Ying, Qifang Wang, Ren-Chao Chen, Qi Shen, Guo-Liang Xu, Jinsong Li, Qiang Sun, Zhi-Qi Xiong, Hui Yang

**Affiliations:** 1 Institute of Neuroscience, State Key Laboratory of Neuroscience, Key Laboratory of Primate Neurobiology, CAS Center for Excellence in Brain Science and Intelligence Technology, Shanghai Institutes for Biological Sciences, Chinese Academy of Sciences, Shanghai 200031, China; 2 State Key Laboratory of Cell Biology, Shanghai Key Laboratory of Molecular Andrology, CAS Center for Excellence in Molecular Cell Science, Institute of Biochemistry and Cell Biology, Shanghai Institutes for Biological Sciences, Chinese Academy of Sciences, Shanghai 200031, China; 3 State Key Laboratory of Molecular Biology, CAS Center for Excellence in Molecular Cell Science, Institute of Biochemistry and Cell Biology, Shanghai Institutes for Biological Sciences, Chinese Academy of Sciences, University of Chinese Academy of Sciences, Shanghai 200031, China; 4 Shanghai University, Shanghai 200444, China; 5 College of Animal Science and Technology, Guangxi University, Nanning, Guangxi 530004, China; 6 Key Lab of Computational Biology, CAS-MPG Partner Institute for Computational Biology, Shanghai Institutes for Biological Sciences, Chinese Academy of Sciences, Shanghai 200031, China; 7 Interdisciplinary Research Center on Biology and Chemistry, Shanghai Institute of Organic Chemistry, Chinese Academy of Sciences, 345 Lingling Road, Shanghai 200032, China; 8 College of Life Sciences, University of Chinese Academy of Sciences, Beijing 100049, China

**Keywords:** complete gene knockout, CRISPR/Cas9, multiple sgRNAs

## Abstract

The CRISPR/Cas9 system is an efficient gene-editing method, but the majority of gene-edited animals showed mosaicism, with editing occurring only in a portion of cells. Here we show that single gene or multiple genes can be completely knocked out in mouse and monkey embryos by zygotic injection of Cas9 mRNA and multiple adjacent single-guide RNAs (spaced 10-200 bp apart) that target only a single key exon of each gene. Phenotypic analysis of F0 mice following targeted deletion of eight genes on the Y chromosome individually demonstrated the robustness of this approach in generating knockout mice. Importantly, this approach delivers complete gene knockout at high efficiencies (100% on *Arntl* and 91% on *Prrt2*) in monkey embryos. Finally, we could generate a complete *Prrt2* knockout monkey in a single step, demonstrating the usefulness of this approach in rapidly establishing gene-edited monkey models.

## Introduction

The CRISPR/Cas9 system has been used to generate genome modification in various species by directly injecting Cas9 mRNA and single-guide RNAs (sgRNAs) into pronuclear stage zygotes, leading to a DNA double-strand break (DSB) at a specified locus^1,2^. The DSB could then be repaired with error-prone non-homologous end joining, resulting in mutant animals carrying frame-shift mutations. However, the majority of gene-edited animals generated by this method showed gene-functional mosaicism, with gene disruption occurring in some cells but not others^3,4^. For phenotypic analysis of gene function, gene-edited animals with mosaicism required further serial crossbreeding to generate complete gene knockout animals. Such a procedure becomes rather laborious when deletion of multiple genes is required. The problem of mosaicism becomes even more serious when the CRISPR/Cas9 method is applied to large animals such as macaque monkeys, which have a long breeding cycle (5-6 years) and a small litter size (1 per birth)^4,5^.

Many previous studies have attempted to produce gene-modified animals without mosaicism in a single step, a procedure especially useful for large animals. These include injection of Cas9 mRNA and sgRNAs into the oocyte rather than the zygote^6^, injection of Cas9 protein^7,8^, or dual sgRNAs injection^9,10^. However, these approaches achieved complete biallelic knockout animals with rather low efficiency.

In this study, we show that single gene or multiple genes can be completely knocked out in all cells of nearly 100% of CRISPR/Cas9-injected mice and monkeys, using multiple sgRNAs. We demonstrate the usefulness of this approach in rapid screening of gene functions in F0 mice and in the rapid generation of monkeys with a complete knockout of a specific gene.

## Results

### Complete deletion of GFP in transgenic GFP embryos

**Figure 1:**
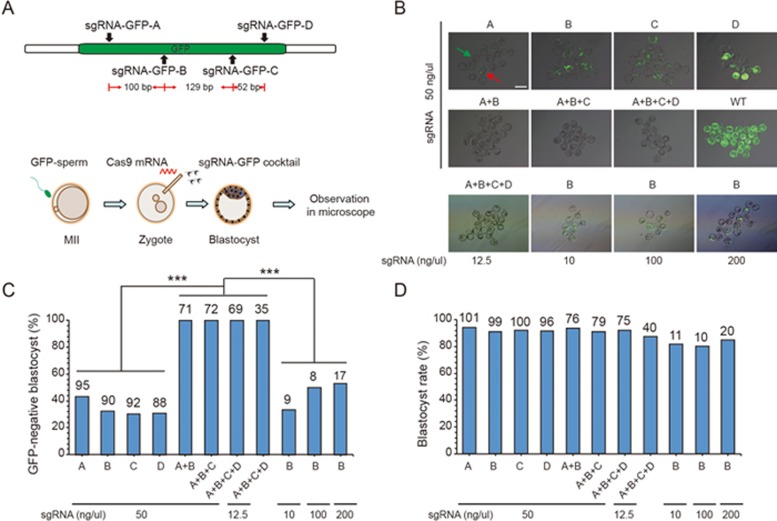
Complete deletion of *GFP* in GFP-embryos by C-CRISPR. **(A)** Schematic diagram of sgRNA-targeting sites at *GFP* locus and experimental design. *Cas9* mRNA and single or multiple sgRNAs-targeting *GFP* were injected into individual mouse zygotes and GFP signal was examined at blastocyst stage. **(B)** Images of GFP signal of blastocysts resulting from different forms of *GFP* targeting. Examples of GFP-negative blastocyst (green arrow, no GFP signal in any cells of the blastocyst) and GFP-positive blastocyst with mosaicism (red arrow, GFP signal in some cells of the blastocyst) are shown in the first image. The concentration of each sgRNA was shown in each group. Scale bar, 200 μm. **(C)** Histograms showing percentages of GFP-negative blastocysts (without mosaicism) resulting from different forms of *GFP* targeting. Two or more targeting sgRNAs (sgRNA-*GFP*-A+B, A+B+C, or A+B+C+D) resulted in higher percentages of GFP-negative blastocysts than single sgRNA targeting (sgRNA-*GFP*-A, B, C, or D). Number above the bar, total number of blastocysts counted (^***^P < 0.001, *χ*^2^ test). The concentration of each sgRNA was shown at the bottom. **(D)** Blastocyst rate of embryos resulting from different *GFP* targeting. Number, total number of zygotes injected. The concentration of each sgRNA was shown at the bottom.

We first tested whether using multiple rather than single sgRNAs could achieve complete gene knockout. To facilitate the estimation of mosaicism, we co-injected *Cas9* mRNA and *GFP*-targeting sgRNAs into zygotes derived from wild-type (WT) female mice mated with homozygous *Actin*-*EGFP* transgenic males (with ubiquitous expression in all cells) and then analyzed *GFP* expression pattern at the blastocyst stage (Figure 1A). We designed four sgRNAs with target sites spaced 10-200 bps at the exon of the *GFP* gene, surmising that such short distances between adjacent sgRNAs could lead to a high frequency of large-fragment exon deletion rather than regular indels, resulting in more frequent complete gene knockout. We found that 31%-44% of the blastocysts derived from zygotes targeted with single-sgRNAs exhibited no GFP signal, and the rest showed mosaicism, with GFP signals in some blastomeres (Figure 1B and 1C). In control embryos without gene editing, all the blastocysts were GFP-positive (Figure 1B and 1C). By contrast, after injection of multiple (2, 3, or 4) sgRNAs that targeted *GFP*, we found that the GFP signal was eliminated in all injected blastocysts, indicating a *GFP* complete knockout (Figure 1B and 1C). Furthermore, multiple-sgRNA targeting showed no obvious deleterious effect on the embryo development which was comparable with embryos obtained by single-sgRNA targeting (Figure 1D).

We next asked whether the sgRNA concentration could affect the efficiency of complete *GFP* knockout. Whereas in the above experiments we had injected sgRNAs at 50 ng/μl for each sgRNA, we now injected 4 sgRNAs (sgRNA-*GFP*-A+B+C+D) at 12.5 ng/μl for each sgRNA, or injected a single sgRNA (sgRNA-*GFP*-B) at 10, 100, and 200 ng/μl, respectively (Figure 1B). We found that all embryos injected with the 4 sgRNAs at this lower concentration exhibited no GFP signal whereas embryos injected with 1 sgRNA at different concentrations showed GFP mosaicism (Figure 1B and 1C). These results indicate that *GFP* complete knockout is not due to the increase of the concentration of sgRNAs, but to multiple-sgRNA targeting.

### Complete deletion of Tyr gene in mice

**Figure 2:**
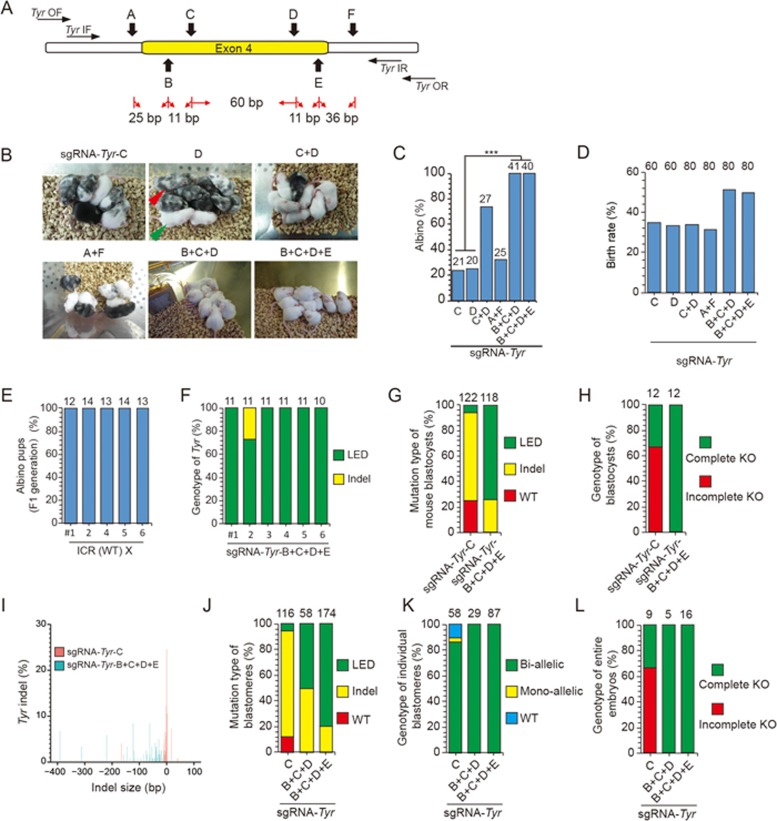
One-step generation of *Tyr* complete knockout mice by C-CRISPR. **(A)** Schematic diagram of sgRNA-targeting sites at *Tyr* locus. The space between each sgRNA is from the end of last base of one sgRNA to the first base of another sgRNA (including PAM sequence). **(B)** Representative results of pigmentation phenotypes of mice resulting from *Tyr* targeting. Green arrowhead, albino; red arrowhead, mosaic pigmentation. **(C)** Histograms showing the percentage of albino mice resulting from *Tyr* targeting. Two or more targeting sgRNAs (sgRNA-*Tyr*-C+D, B+C+D, or B+C+D+E) resulted in higher percentage of albino mice than single-sgRNA targeting (sgRNA-*Tyr*-C or D). Number, total number of mice counted (^***^P < 0.001, *χ*^2^ test). **(D)** Birth rate of mice resulting from *Tyr* targeting. Number, total number of embryos transferred. **(E)** Reproductive ability and germline transmission of *Tyr* knockout mice by C-CRISPR. Five gene-modified mice (sgRNA-*Tyr*-B+C+D+E) were mated with WT ICR mice individually. Each pair of mice gave birth to two litters of pups. **(F)** Albino mice resulting from sgRNA-*Tyr*-B+C+D+E targeting were sequenced. DNA was isolated from tails of albino mice and PCR amplified. #2 mice contained large-fragment exon deletion and indels. LED, large-fragment exon deletion; Indels, insertion, or deletion of bases. Number above each column, total TA clones sequenced. **(G** and **H)** Percentages of different mutation types **(G)** and genotypes **(H)** of blastocysts resulting from *Tyr* targeting. Number, total TA clones sequenced and analyzed. WT, wild-type allele. Complete KO, blastocyst with complete knockout mutations; Incomplete KO, blastocyst with WT allele and knockout mutations. Number, total blastocysts counted. **(I)** Size distribution of deletions or insertions in blastocysts with *Tyr* targeting. **(J** and **K)** Percentages of different mutation types **(J)** and genotypes **(K)** for individual blastomeres of 8- to 16-cell embryos with *Tyr* targeting. LED, large-fragment exon deletion; Bi-allelic, bi-allelic mutations; Mono-allelic, mono-allelic mutations. Number, total alleles, or blastomeres analyzed. **(L)** Percentages of different genotypes of entire 8- to 16-cell embryos with *Tyr* targeting. Number, total embryos analyzed.

To further test whether an endogenous gene with two alleles could also be completely knocked out by this approach, we targeted the *Tyrosinase* gene (*Tyr*, for pigmentation) at the zygote stage, and produced gene-edited mice via implantation of two-cell embryos (Figure 2A). We found that targeting a single exon with 3 or 4 sgRNAs (sgRNA-*Tyr*-B+C+D or sgRNA-*Tyr*-B+C+D+E) resulted in 100% of albino mice (Figure 2A-2C), indicating complete *Tyr* knockout in all gene-edited mice. By contrast, 1 sgRNA or 2 sgRNAs targeting one exon (sgRNA-*Tyr*-D or sgRNA-*Tyr*-C+D), or 2 sgRNAs targeting two introns flanking one exon (sgRNA-*Tyr*-A+F) resulted in 26%-77% of gene-edited mice with mosaicism of pigmentation (Figure 2A-2C). Furthermore, multiple-sgRNA targeting showed no obvious deleterious effect on either embryo development or birth rate of gene-edited mice, compared with single-sgRNA targeting (Figure 2D). The gene-modified mice created by 4-sgRNA targeting (sgRNA-*Tyr*-B+C+D+E) showed normal reproductive ability when mated to ICR (albino) mice and all the progeny were albino, indicating complete knockout of *Tyr* in all germ cells of these gene-modified mice (Figure 2E).

DNA genotyping analysis of the *Tyr* gene-edited embryos (*n* = 12) and mice (*n* = 6) created by 4-sgRNA targeting (sgRNA-*Tyr*-B+C+D+E) confirmed *Tyr* complete knockout. It also revealed that with an increasing number of sgRNAs used, the percentage of gene-edited mice exhibiting large-fragment exon deletion (defined as > 30 bp in this work) rather than regular indels (defined as ≤ 30 bp)^11^ at the target locus gradually increased (Figure 2F-2I; Supplementary information, Figure S1A-S1E). We further examined the gene-edited 8- to 16-cell embryos at the single-cell level. We found that all blastomeres created by 3- to 4-sgRNA targeting (*n* = 116) carried bi-allelic mutations, resulting in complete *Tyr* knockout in all embryos (*n* = 21) (Figure 2J-2L; Supplementary information, Figure S1F and S1G). By contrast, blastomeres with single-sgRNA targeting (*n* = 58) showed 10.3% of WT alleles and 3.4% of mono-allelic mutations, resulting in 67% of embryos (6/9) with *Tyr* incomplete knockout (Figure 2J-2L; Supplementary information, Figure S1F-S1H). Together, these results indicate that a single allele or two alleles of gene could be completely knocked out in the embryo by a cocktail of targeting sgRNAs in the CRISPR/Cas9 system (C-CRISPR hereafter), when a single exon is targeted with two or more closely spaced sgRNAs. Such high efficiency in complete gene knockout appears to be caused by simultaneous frame-shift mutation and exon deletion.

### One-step generation of complete triple knockout

**Figure 3:**
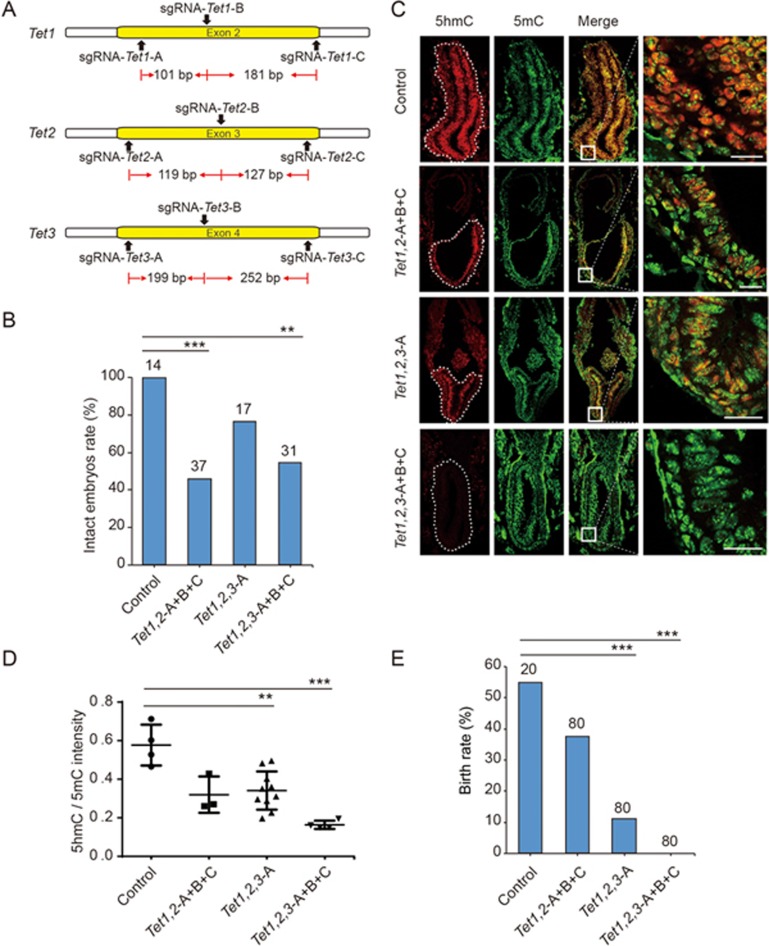
One-step generation of mice with complete triple knockout of *Tet* genes by C-CRISPR. **(A)** Schematic diagram of sgRNA-targeting sites at *Tet1*, *Tet2* and *Tet3* loci, with three sgRNAs for each locus. **(B)** Percentages of control and *Tet* gene-edited E7.5 embryos that were intact. Number, total number of embryos counted (^***^P < 0.001, ^**^P < 0.01, *χ*^2^ test). **(C)** Immunostaining of tissue sections showing the level of 5hmC (red) and 5mC (green) in E7.5 embryos with different forms of *Tet* gene targeting. White-dashed line, epiblast of E7.5 embryos, with boxed regions shown at a higher resolution on the right. “5hmC”, 5-hydroxymethylcytosine; “5mC”, 5-methylcytosine; scale bar, 50 μm. **(D)** The relative levels of 5mC and 5hmC in E7.5 embryos. Each data point represents the ratio (5hmC/5mC) of the average immunofluorescence intensities measured from one tissue section. Error bars, SEM (^***^P < 0.001, ^**^P < 0.01, unpaired *t*-test). **(E)** Birth rate of mice for control embryos and different forms of *Tet* gene targeting. Number: total number of embryos transferred to surrogates (^***^P < 0.001, *χ*^2^ test).

To test whether multiple genes could be completely knocked out in one step by C-CRIPSR injection of the zygotes, we simultaneously targeted *Tet1*, *Tet2*, and *Tet3*, three functionally redundant genes involved in DNA oxidation^12^ (Figure 3A). After embryo implantation, we harvested E7.5 embryos and examined the presence of *Tet* genes by assaying 5-hydroxymethylcytosine (5hmC) level, the main product derived from oxidation of 5-methylcytosine (5mC) by Tet proteins. In *Tet* gene-edited embryos (Group I: *Tet1* and *Tet2* double targeting by 6 sgRNAs, *Tet1, 2*-A+B+C; Group III: *Tet1*, *Tet2*, and *Tet3* triple targeting by 9 sgRNAs, *Tet1, 2, 3*-A+B+C), about half had degenerated by E7.5 (Figure 3B), consistent with previous reports^13,14,15,16^. Immunostaining of epiblast tissue sections for 5hmC and 5mC in E7.5 embryos showed a high level of 5hmC in all cells of WT embryos (Figure 3C). By contrast, embryos with *Tet1*, *Tet2*, and *Tet3* triple targeting by 3 sgRNAs (Group II: one sgRNA for each gene, *Tet1, 2, 3*-A) showed a mosaic pattern of 5hmC staining, with high level of 5hmC in some cells (Figure 3C). In addition, embryos with *Tet1* and *Tet2* double targeting by 6 sgRNAs (3 sgRNAs for each gene) showed similar mosaic staining of 5hmC. However, embryos with *Tet1*, *Tet2*, and *Tet3* triple targeting by 9 sgRNAs (3 sgRNAs for each gene) showed complete absence of 5hmC (Figure 3C), indicating complete deletion of all three *Tet* genes. The average ratio of 5hmC/5mC was quantified by measuring the intensity of immunofluorescence, and the results showed that the ratio dropped in half in Group I & II and to much lower levels in Group III (Figure 3D). Finally, double-*Tet*-knockout embryos created with 6 sgRNAs showed reduced birth rate, and triple *Tet*-knockout embryos created by 9 sgRNAs yielded no live birth (Figure 3E), consistent with previous studies^13,14,15,16^. These results demonstrate the efficiency of one-step deletion of multiple genes using the C-CRISPR method.

### Phenotype analysis of F0 mice with complete gene knockout by C-CRISPR

**Figure 4:**
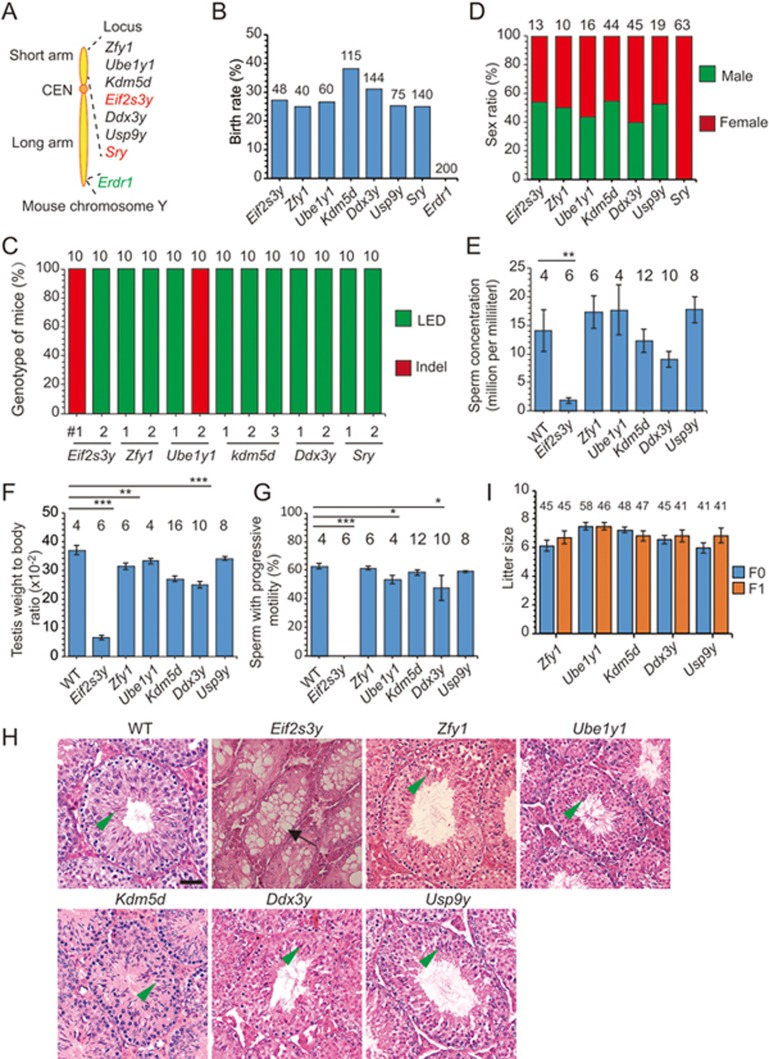
Phenotype analysis of F0 mice with individual Y chromosome gene deletion by C-CRISPR. **(A)** Schematic diagram of eight targeted genes in Y chromosome. Two sgRNAs were designed to target each gene. Red, gene previously studied with conventional knockout mice; green, gene located in both X and Y chromosomes. **(B)** Birth rate of mice with embryos edited by the C-CRISPR method for deletion of individual Y-chromosome gene. Number, total embryos transferred. *Erdr1* targeting resulted in embryonic lethality. **(C)** Sex ratio of mice with deletion of different Y-chromosome genes. **(D)** DNA sequence analysis of gene-edited mice in Y chromosome by C-CRISPR. Tails of mice resulting from different gene targeting in Y chromosome were genotyped. LED, large-fragment exon deletion; Indels, insertion, or deletion of bases. Number above each column, total TA clones sequenced. (**E**-**G**) Sperm concentration **(E)** weight-to-body ratio of testis **(F)** and percentages of sperm with progressive motility **(G)** for mice with deletion of different Y-chromosome genes. Number, total number of samples counted (^***^P < 0.001, ^**^P < 0.01, ^*^P < 0.05, *χ*^2^ test). **(H)** Histological analysis of the testis sections from adult mice with deletion of different Y-chromosome genes. Arrow, abnormal vacuoles of seminiferous tubule; arrowhead, sperm. Abnormality was found only in the *Eif2s3y* deleted testis. Bar, 20 μm. **(I)** Reproductivity of F0 and F1 mice with deletion of different Y-chromosome genes. Number, total pups obtained.

We next employed the C-CRISPR method for phenotypic analysis of the function of various Y-chromosome genes, and tested the efficiency of this one-step complete gene knockout in mice for rapid functional screening of a large number of genes. The Y chromosome is highly specialized for male sex differentiation and fertility^17,18^. However, so far only a few genes have been assessed for biological function in targeted knockout mice. We decided to target eight individual single-copy Y chromosome genes (Figure 4A). We found that embryos targeted at *Erdr1*, located in both Y and X chromosomes, resulted in embryonic lethality (Figure 4B). Deletion of the other seven genes individually had no apparent effect on the normal birth rate. Genotyping these mice confirmed the complete deletion of the individual genes (Figure 4C; Supplementary information, Table S1). Consistent with previous reports^19,20,21^, deletion of *Sry* resulted in sex reversal of male embryos (Figure 4D) and male mice targeted for *Eif2s3y* deletion were infertile and had hypoplastic testes (Figure 4E-4H). By contrast, all male mice with deletions for each of the other five genes (*Zfy1, Ube1y1, Kdm5d, Ddx3y, Usp9y*), and their F1 male offspring, were fertile and showed normal reproductive ability (Figure 4E-4I). Taken together, these results showed that the C-CRISPR method could be applied for the rapid phenotypic analysis of gene functions by generating complete gene knockout mice in F0.

### C-CRISPR-mediated complete gene knockout in monkeys

**Figure 5:**
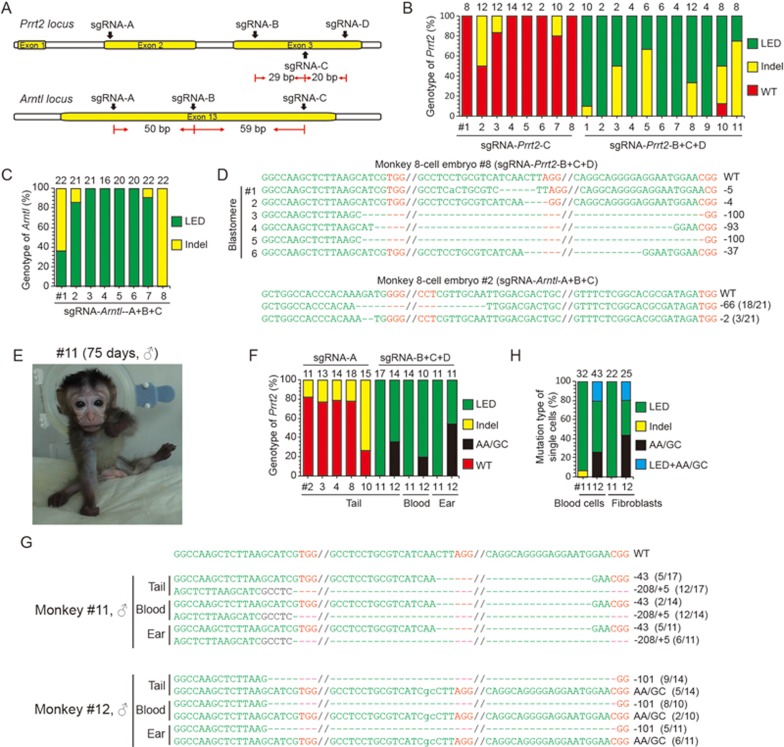
One-step generation of complete knockout monkey by C-CRISPR. **(A)** Schematic diagram of sgRNA-targeting sites at *Prrt2* and *Arntl* loci in monkey. **(B** and **C)** Percentages of different mutation types for 8-cell embryos with *Prrt2* **(B)** and *Arntl* **(C)** targeting. LED, large-fragment exon deletion. Indels, insertion, or deletion of bases. Number above each column, total alleles analyzed from blastomeres of 8-cell embryos with *Prrt2* targeting or total TA clones sequenced from 8-cell embryos with *Arntl* targeting. **(D)** Genotyping analysis on single blastomeres of 8-cell embryos targeted with sgRNA-*Prrt2*-B+C+D or whole 8-cell embryos targeted with sgRNA-*Arntl*-A+B+C. The sgRNA targeted sequences are labeled in green and the PAM sequences are labeled in red; deleted nucleotides are indicated by hyphens. Dashed lines mark the region omitted for clarity. **(E)** Photograph of *Prrt2*-knockout monkey (#11). **(F)** Percentages of different mutation types found in tail, ear, and blood cells from aborted and live monkeys by *Prrt2* targeting. Aborted monkeys with sgRNA-*Prrt2*-A targeting: monkey #2, #3; live monkeys with sgRNA-*Prrt2*-A targeting: monkey #4, #8, #10; live monkeys with sgRNA-*Prrt2*-B+C+D targeting: monkey #11, #12. AA/GC, a point mutation in *Prrt2*(335N to 335A). **(G)** Representative sequences from ear, tail, and blood cells of monkey #11 and #12 with sgRNA-*Prrt2*-B+C+D targeting. The sgRNA-targeting sequences are labeled in green and PAM sequences are labeled in red; deleted nucleotides are indicated by hyphens. Dashed lines mark the region omitted for clarity. **(H)** Single-cell analysis on blood cells and fibroblasts from two live monkeys with *Prrt2* editing (#11, #12). Number, total number of cells analyzed.

We next examined whether the C-CRISPR method could be used to generate monkeys with complete gene knockouts. We performed targeted deletion of *Prrt2* (mutations in paroxysmal kinesigenic dyskinesia), and *Arntl* (a core component of the circadian clock) in the cynomolgus monkey (*Macaca fascicularis*) (Figure 5A). After pretesting the DNA cleavage efficiencies of each sgRNA in monkey embryos or monkey COS-7 cells (Supplementary information, Figure S2A and data not shown), we targeted monkey embryos with 3 sgRNAs of *Prrt2* having relatively higher cutting efficiencies (sgRNA-*Prrt2*-B+C+D). When all blastomeres of each individually injected 8-cell embryos were analyzed, we found that, with the exception of one blastomere that carried mono-allelic mutation, all blastomeres with detectable PCR signals (*n* = 33, from 11 embryos) carried bi-allelic mutations in *Prrt2* gene, resulting in 10 complete knockout embryos out of 11 injected embryos (Figure 5B and 5D, Supplementary information, Figure S2B). By contrast, control 8-cell blastomeres from 8 embryos injected with a single sgRNA (sgRNA-*Prrt2*-C) showed 90% of WT alleles and 10% of mono-allelic mutations, resulting in 3 incomplete knockout embryos and 5 WT embryos (Figure 5B; Supplementary information, Figure S2B and S2C). For *Arntl* targeting, we found that all eight examined embryos targeted with 3 sgRNAs (sgRNA-*Arntl*-A+B+C) showed complete knockout of *Arntl* (Figure 5C and 5D; Supplementary information, Figure S2D).

To generate *Prrt2* knockout monkeys, we transferred 29 embryos targeted with 3 sgRNAs (sgRNA-*Prrt2*-B+C+D) into nine surrogates and obtained one pregnant monkey, which gave birth of two live male monkeys (Figure 5E). Further DNA sequencing analysis of the ear and tail tissues as well as blood cells from these two monkeys showed that one monkey (#11) carried complete *Prrt2* knockout and the other (#12) an incomplete knockout of *Prrt2*, with a large-fragment exon deletion and point mutations (335N to 335A) (Figure 5F to 5H; Supplementary information, Figure S2E to S2G). By contrast, after 55 embryos injected with a single sgRNA (sgRNA-*Prrt2*-A) were transferred, we obtained six stillborns and four live monkeys. Two of the stillborns (#2 and #3) and three live monkeys (#4, #8 and #10) carried varying extents (20%-60%) of deletion of *Prrt2* and the remaining five monkeys exhibited no gene editing (Figure 5F). These results suggest the C-CRISPR method could achieve essentially complete gene knockout in monkeys.

### Off-target effect in complete knockout animals by C-CRISPR

Finally, we examined whether multiple-sgRNA targeting by the C-CRISPR method induces more off-target effects than single-sgRNA targeting. As previously described^22^, we examined up to 10 off-target sites for each sgRNA in six mice derived from a 4-sgRNA-targeting mutagenesis (*Tyr*-B+C+D+E-#1, #2, #3, #4, #5, #6), and two monkeys from 3-sgRNA targeting (*Prrt2*-B+C+D-#11, #12). DNA sequencing of the PCR products amplified from these genomic sites showed that no mutations occurred at any of these loci (Supplementary information, Figure S3A and S3B). We also performed whole-genome sequencing on these samples, as well as on two control monkeys from single-sgRNA targeting (*Prrt2*-A-#8, #9) at a sufficient depth to detect off-target mutations (15 × for the mice and 25 × for the monkeys). We then searched for off-target effects in the genome allowing up to five mismatches of 4 sgRNAs in the mice (*Tyr*-B, C, D, and E) and 3 sgRNAs (*Prrt2*-B, C, and D) in the monkeys (Table 1; Supplementary information, Table S2 and S3). Of 10 201 and 19 447 possible off-target sites in mice and monkeys, respectively, we found no indels in the six mice and monkey #12 after filtering variants shared in individual samples as done in previous studies^23,24^ (Table 1; Supplementary information, Table S3). For monkey #11, we observed indels in three off-target sites, with five mismatches in both PAM-distal and PAM-proximal regions (Supplementary information, Table S3). We further examined the three off-target sites in monkey #11 by PCR amplification and sequencing, and found that one site was not a true off-target site (Supplementary information, Figure S3C and S3D) and the other two sites were associated with a repeated DNA sequence, which could not be distinguished by sequencing (Supplementary information, Table S3). We also examined genomic rearrangements, including deletions, duplications, inversions, and copy number variations, using the similar strategies, and found no rearrangements in the six mice and two monkeys (#11 and #12) (Supplementary information, Table S3). Thus C-CRISPR approach did not induce significant off-target alterations in gene-edited mice and monkeys beyond that expected for CRISPR/Cas9-mediated editing in general^1,24,25,26^.

## Discussion

The CRISPR/Cas9 system is an efficient gene-editing method, but the majority of gene-edited animals show mosaicism that impedes effective phenotypic analysis of the consequence of gene deletion. In this study, we found that a single gene or multiple genes could be efficiently deleted in all cells of nearly 100% of CRISPR/Cas9-injected mice and monkeys, using a modified “C-CRISPR” approach. Such high efficiency in complete gene knockout appears to be caused by simultaneous frame-shift mutation and exon deletion.

There are several distinct features of the C-CRISPR method that ensure efficient gene deletion. First, when using multiple sgRNAs to target a single exon of a gene for which there is only one allele, such as genes in Y chromosome, 2 to 3 sgRNAs are required; for bi-allelic autosomal genes, 4 to 6 sgRNAs are required. Second, the close apposition of DNA exon sites targeted by multiple sgRNAs is a key feature of our approach. Third, it is important to target a key exon of the ORF of the gene^27^ because the change of only one or two amino acids in a key domain of a protein may completely disrupt its function. Finally, we noted the importance of pretesting the efficiency of sgRNAs in cleaving DNAs in embryos, especially for gene editing in the monkey because the majority of sgRNAs were not efficient. Together, these features promote large-fragment exon deletion in addition to regular indels, leading to complete gene knockout. We summarized a protocol for the application of C-CRISPR in mice and monkeys (Supplementary information, Data S1).

We also performed comprehensive off-target analysis on the animals produced by multiple-sgRNA targeting and found no obvious off-target effects. This is consistent with previous studies that off-target mutations are rare in Cas9-modifed animals produced by dual-sgRNA targeting^23^. Our selection of sgRNAs with minimal off-target effects, based on the online software (http://crispr.mit.edu/ and http://www.rgenome.net/cas-offinder) could have contributed to the decrease of off-target mutations.

Recently, other strategies have been used to reduce mosaic mutations in the mouse and monkey^28,29^. By electroporation of Cas9 protein/sgRNA into *in vitro* fertilized zygotes at the early pronuclear stage, Hashimoto *et al*. generated non-mosaic mutants at two gene loci in the mouse^28^. However, more gene loci need to be tested and whether this approach also works in other species, such as non-human primates, needs to be proven, since the DNA cleavage efficiency of sgRNA is much lower in monkey embryos than in mouse embryos^2^. Tu *et al*. reported that promoting Cas9 degradation could also reduce mosaic mutations in non-human primate embryos, resulting in 28.97% monkey embryos with mono-allelic or bi-allelic mutations in all cells at one gene locus^29^. In our study, we have shown that the C-CRISPR approach produces complete gene knockout monkey embryos with much higher efficiency (100% for *Arntl* and 91% for *Prrt2* achieving bi-allelic mutations in all cells). Our approach with such high efficiency could markedly reduce the number of animals needed for experiments. This is highly desirable from the ethical viewpoint, especially when using non-human primates as animal models. Meanwhile, it would be interesting to combine our multiple sgRNAs strategy with Cas9 protein in early pronuclear zygotes or modified ubiquitin-Cas9 mRNA to generate animals carrying the same genetic mutation in future studies.

The C-CRISPR approach can be particularly useful for large-scale (10-100 genes) functional screening in F0 mice. For example, a large number (> 20) of single-gene knockout F0 mice could be obtained by a single round of injection of ∼ 100 zygotes and three rounds of such injections may be carried out in a day by one person. Another application of this approach in mice is to generate complete gene-knockout blastocysts for the purpose of interspecies blastocyst complementation, which is at present not feasible but highly desirable^30^. Finally, the C-CRISPR approach also offers a more efficient one-step process for studying gene function in monkey and human embryos. In this study, we were able to obtain one complete gene knockout monkey out of two live monkeys produced by this method, whereas no complete knockout monkey was obtained out of 10 monkeys produced by the single-sgRNA-targeting method. Furthermore, our monkey embryo data also indicate that this approach will make genetic studies in monkeys much more realistic in the future.

## Materials and Methods

### Animal ethics statement

The use and care of animals complied with the guideline of the Biomedical Research Ethics Committee at the Shanghai Institutes for Biological Science, Chinese Academy of Science (CAS), which approved the application entitled “Reproductive physiology of cynomolgus monkey and establishment transgenic monkey” (#ER-SIBS-221106P).

### Production of Cas9 mRNA and sgRNA

The T7 promoter was added to the *Cas9* coding region by PCR amplification of px260, using primer *Cas9* F and R (Supplementary information, Table S4). The *T7-Cas9* PCR product was purified and used as the template for *in vitro* transcription (IVT) using mMESSAGE mMACHINE T7 ULTRA kit (Life Technologies). The T7 promoter was added to the sgRNA template by PCR amplification of px330, using primers listed in (Supplementary information, Table S4). The T7-sgRNA PCR product was purified and used as the template for IVT using MEGA shortscript T7 kit (Life Technologies). Both the *Cas9* mRNA and the sgRNAs were purified using the MEGA clear kit (Life Technologies) and eluted in RNase-free water.

### Zygote injection, embryo culturing, and embryo transplantation

For mice gene editing, super ovulated female C57BL/6 mice (3 weeks old) or B6D2F1 (C57BL/6 × DBA2J) mice (7-8 weeks old) were mated to C57BL/6 or B6D2F1 males, and fertilized embryos were collected from oviducts. For *GFP* targeting, zygotes were harvested after mating homozygous *Actin-GFP* transgenic^31^ male mice with WT female mice. Cas9 mRNA (50 ng/μl) and sgRNAs (50 ng/μl for each sgRNA in most of experiments and 20 ng/μl for each sgRNA in *Tet1, 2, 3* triple-gene knockout experiments) were mixed and injected into the cytoplasm of fertilized eggs with well recognized pronuclei in a droplet of HEPES-CZB medium containing 5 μg/ml cytochalasin B (CB) using a FemtoJet microinjector (Eppendorf) with constant flow settings. The injected zygotes were cultured in KSOM medium with amino acids at 37 °C under 5% CO_2_ in air until the 2-cell stage by 1.5 days. Thereafter, 25-30 2-cell embryos were transferred into oviducts of pseudopregnant ICR females at 0.5 dpc.

For monkey gene editing, laparoscopy was used for oocyte collection. Oocytes were aspirated from follicles 2-8 mm in diameter, about 32-36 h after hCG stimulation^32^. The collected oocytes were cultured in the pre-equilibrated maturation medium. Metaphase II arrested oocytes were used to perform intracytoplasmic sperm injection, and fertilization was confirmed by the presence of two pronuclei. The zygotes were injected with *Cas9* mRNA (100 ng/μl) and sgRNAs (50 ng/μl for each sgRNA). After injection, the embryos were cultured in HECM-9 medium until they were transferred to surrogates on the next day. Some embryos were cultured until the morula/blastocyst stage and harvested for genome extraction and analysis.

### Single-cell PCR analysis

For picking up and transferring single cells, we used a glass capillary under a dissection microscope. Mouse or monkey 8-16 cell stage embryos were digested with acid Tyrode solution to remove the zona pellucida. Then the embryos were transferred into 0.25% trypsin and the individual blastomeres were separated by gentle pipetting. Finally, the blastomeres were washed in 0.25% trypsin for 7 to 10 times and transferred into a PCR tube. Fibroblast or white blood cells were then diluted stepwise with KSOM until the cells were fully dispersed. Following 7-10 washes with KSOM, a single cell was transferred into a PCR tube. An aliquot of 1.5 μl lysis buffer containing 0.1% tween 20, 0.1% Triton X-100 and 4 μg/ml proteinase K was then pipetted into the tube. Each tube was centrifuged to facilitate mixing. The lysis was performed at a temperature of 56 °C for 30 min, followed by 95 °C for 5 min. The products of the lysis program were used as templates in a nested PCR analysis. All operations were carried with care to prevent contaminating the samples.

### Genotyping

The genotypes of mutant mice were determined by PCR of genomic DNA extracted from tails. ExTaq was activated at 95 °C for 3 min, and PCR was performed for 34 cycles at 95 °C for 30 s, 62 °C for 30 s, and 72 °C for 1 min, with a final extension at 72 °C for 5 min. To amplify DNA from blastocysts, single blastocysts were washed six times with KSOM before they were transferred directly into PCR tubes containing 1.5 μl embryo lysis buffer (0.1% tween 20, 0.1% Triton X-100 and 4 μg/ml proteinase K) and incubated at 56 °C for 30 min, heat inactivated with proteinase K at 95 °C for 10 min. PCR ampliﬁcation was performed using nested primer sets. ExTaq was activated at 95 °C for 3 min, and PCR was performed for 34 cycles at 95 °C for 30 s, 62 °C for 30 s, and 72 °C for 1 min, with a final extension at 72 °C for 5 min. Secondary PCR was performed using 0.5 μg primary PCR product and nested inner primer. PCR was carried out in the same reaction mixture. The PCR product was gel purified and cloned using a pMD-19t cloning kit (Takara) following the manufacturer's instructions. Colonies were picked from each transformation and then Sanger sequencing was applied to detect mutations.

### HE staining and immunostaining

To analyse testis, part of the tissue was fixed in Bouin's solution overnight. The fixed tissues were then embedded into paraffin, and 5-m sections were cut with a Microtome. After being deparaffinized, and rehydrated, sections were stained with hematoxylin-eosin. For 5hmC staining, post-implantation embryos were dissected from pregnant females at the day indicated. Then embryos were fixed with 4% paraformaldehyde, embedded in OCT compound (Sakura) and 8 μm sections were cut. After washing with PBS, the sections were treated with hydrochloric acid solution (4N hydrochloric acid, 0.1% Triton X-100 in distilled water), followed by washing with PBS and permeabilization with blocking buffer (1% BSA, 10% goat serum, PBS containing 0.3% Triton X-100). Next, sections were incubated with primary antibodies (mouse anti-5mC antibody, 1:500, Eurogentec#BI-MECY-0100; rabbit anti-5hmC antibody, 1:1 000, Active Motif #39792) overnight at 4 °C and secondary antibodies at room temperature for 1 h. Finally, the slides were mounted in Anti-fade Reagent (Invitrogen) and imaged using a LEICA TCS SP5 II confocal microscope. The signal intensity was determined using Leica Application Suite software. Note, for embryos with *Tet1, 2, 3* triple KO, we used the background (non-specific) for 5hmC to calculate the average ratio of 5hmC/5mC.

### Computer-assisted sperm analysis

This analysis was carried out as described in a previous study^33^. Sperm were diluted to 3-6 × 10^6^ spermatozoa/ml in a buffered solution (NaCl 7.0 g/l, KCl 149 mg/l, CaCl_2_.2H_2_O 147 mg/l, NaHCO_3_ 2.1 g/l, MgSO_4_.7H_2_O 296 mg/l, NaH_2_PO_4_.H_2_O 49.7 mg/l, Glucose 1 g/l, Na pyruvate 121 mg/l, Sucrose 6.63 g/l, TAPSO (buffer) 2.59 g/l (10 mM), Penicillin 57 mg/l, Streptomycin 29 mg/l, pH = 7.3∼7.4) and warmed on a 37 °C plate for 20 min. Sperm suspensions were gently mixed before measuring motility. For each motility measurement, a 5 μl aliquot of sperm suspension was loaded by capillary action using a large-bore pipette tip into the counting chamber (depth, 10 μm) of a pre-warmed Leja slide and analyzed using a computer-assisted semen analysis machine (HTM-TOX IVOS sperm motility analyzer, Animal Motility, version 12.3A; Hamilton Thorne Research). The magnification was 10×. All samples were analyzed at least twice to discard errors due to incorrect sampling. At least five fields per sample were acquired, recording at least 100 motile sperm. Image sequences were saved and subsequently analyzed.

### Whole-genome sequencing and off-target analysis

Whole-genome sequencing was carried out using the Illumina HiSeq X Ten at mean coverages of 15× (sgRNA-*Tyr*-B+C+D+E-#1, #2, #3, #4, #5, #6) and 25× (sgRNA-*Prrt2*-B+C+D-#11, #12). Qualified reads were mapped to the mouse reference genome (mm10) and the assembly Macaca fascicularis genome (v5) by speedseq^34^ (https://github.com/hall-lab/speedseq) with default parameters. All of the mapped data is available from the SRA under accession SRP092889. Potential off-target sites of sgRNAs were predicted as previously reported^35^ (http://www.rgenome.net/cas-offinder/). We extracted all the off-target sites with no more than 5 mismatches for each sgRNA. FreeBayes^36^ (v0.9.10) and LUMPY^37^ (https://github.com/arq5x/lumpy-sv) were run on the aligned sequence files (BAM files) for short indel detection and structural variation discovery. Next, we downsized the short indel variations to the predicted off-target sites, these short indels were then filtered to remove variations overlapped in our samples and the remaining variations were manually inspected by mapping sequencing reads in these regions to the extracted reference sequences with BLAST for potential indel patterns. Finally, variants associated with repetitive DNA sequence were also discarded as these cannot be distinguished from the large number of naturally occurring variants in repetitive DNA sequence in individual animals. For the structural variations, the same strategies were used except that the searching regions were broadened to the 250bp up- and down-stream of the potential off-target sites. Variations after each filtering step were listed in the Supplementary information, Table S3.

### Statistics

*P* values were determined by Student's *t*-test or *χ*^2^ test for all measurements. All error bars denote SEM.

## Author Contributions

EZ designed and performed mouse experiments. YW and DL performed mouse embryo injection. B-AW and LS performed *Tet1, 2, 3* triple knockout experiments. Y-JC and ZL performed monkey *Prrt2* gene knockout experiments. KL performed monkey behave test. Y-JC and J-WL performed *Prrt2* gene knock out monkey genotyping analysis. ZL, YW, Y-HN, and C-CZ performed monkey oocytes collection and embryos transfer experiments. WW and XH performed HE staining and reproductive analysis. XH, XY, and XW performed mice mating and genotyping. WY and QW transferred embryos. YS performed data analysis on off-target effects. G-LX helped with the design of *Tet* gene deletion experiments and provided comments on the manuscript. JL helped with the design of *GFP* and *Tyr* gene deletion experiments. ZX and QS designed the experiments of *Prrt2* knockout in monkey. HY supervised the project, designed experiments and wrote the paper with other helps.

## Competing Financial Interests

The authors declare no competing financial interests.

## Figures and Tables

**Table 1:** Summary of variants detected by whole-genome sequencing

sgRNA	No. of Mismatches	No. of NGG genomic sites^c^	No. of NAG genomic sites^c^	No. of off-target sites					
Monkey				#11			#12		
	0	3	0	NA			NA		
	1	0	0	NA			NA		
*Prrt2*-B+C+D	2	5	13	0			0		
	3	108	81	0			0		
	4	1 037	919	0^a^			0^a^		
	5	8 305	8 976	0^b^			0^a^		
Mouse				#1	#2	#3	#4	#5	#6
	0	4	0	NA	NA	NA	NA	NA	NA
	1	1	0	0	0	0	0	0	0
*Tyr*-B+C+D+E	2	2	2	0	0	0	0	0	0
	3	36	37	0	0	0	0	0	0
	4	394	478	0^a^	0	0^a^	0	0	0
	5	4 357	4 890	0	0	0	0	0	0

^a^ Filtering variations overlapped in individual samples.

^b^ Filtering variations overlapped in individual samples and repeated DNA sequence (> 50 bp).

^c^ The sum of NGG and NAG off-target sites from three sgRNAs (*Prrt2*-B, C, and D) in monkeys and four sgRNAs in mice (*Tyr*-B, C, D, and E).

## Supplementary Information

Supplementary information, Figure S1 Genotyping analysis of gene-edited mice by C-CRISPR.

Supplementary information, Figure S2 Genotyping analysis of genegene-edited edited edited monkey embryos and tissues by C-CRISPR.

Supplementary information, Figure S3 Off-target analysis of mice resulting from *Tyr* targeting and monkeys resulting from *Prrt2* targeting.

Supplementary information, Table S1 Phenotypic analysis of F0 mice with Y-chromosome gene deletions generated by C-CRISPR

Supplementary information, Table S2 Summary of HiSeq X Ten sequencing

Supplementary information, Table S3 Summary of genomic rearrangements, off-target effect and filtering steps

Supplementary information, Table S4 sgRNA sequences and primers sgRNA sequences

Supplementary information, Data S1 C-CRISPR-mediated complete gene knockout in mice and monkeys.
