# Supplementary material for: One-step generation of complete gene knockout mice and monkeys by CRISPR/Cas9-mediated gene editing with multiple sgRNAs
Source: Cell Res. 2017 Jun 6;27(7):933–45. doi: 10.1038/cr.2017.81 (PMC5518993; doi:10.1038/cr.2017.81)
Supplement: Supplementary information, Figure S1 — Genotyping analysis of gene-edited mice by C-CRISPR. [file cr201781x1.pdf]

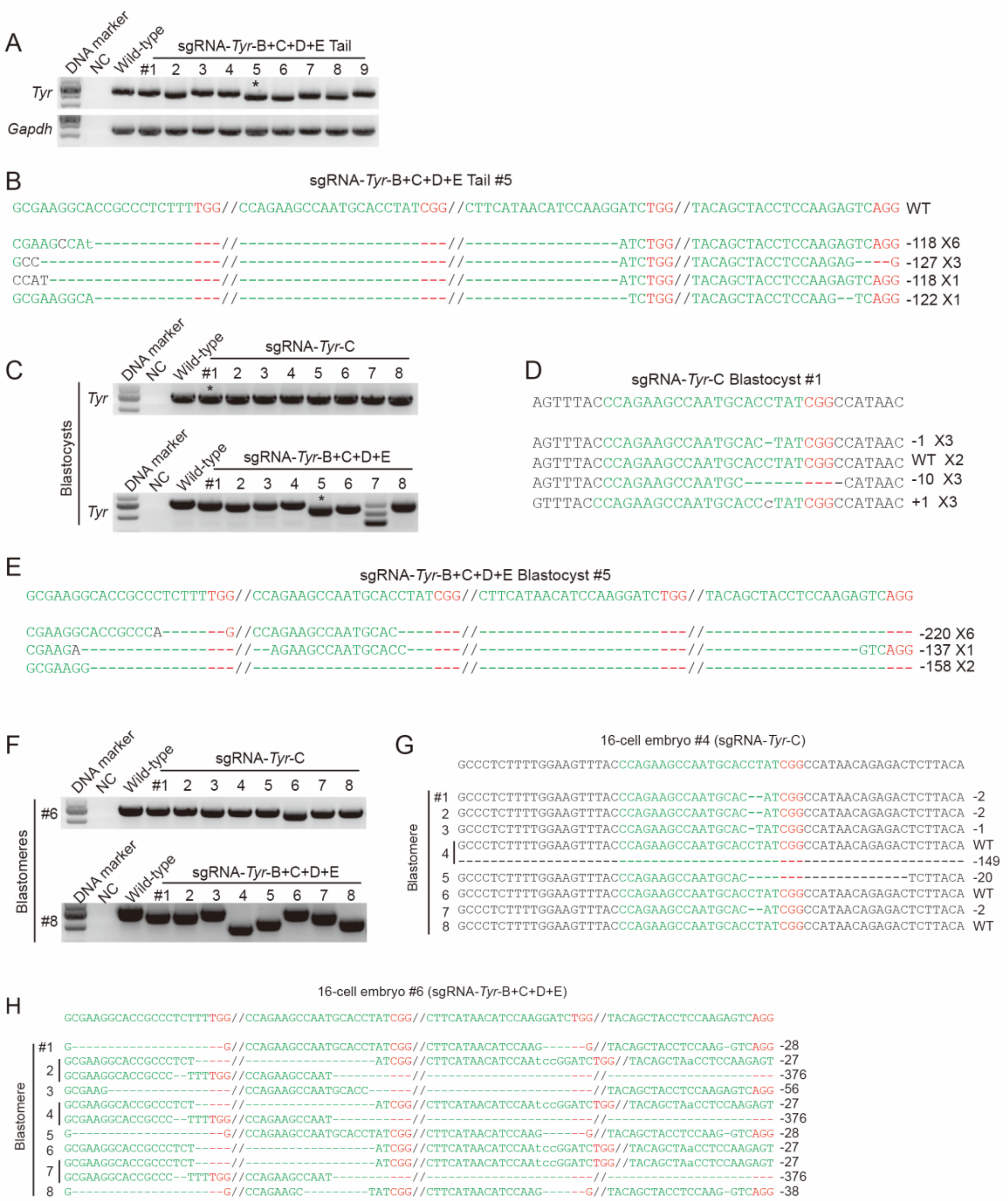
A
DNA marker
NC
Wild-type
sgRNA-Tyr-B+C+D+E Tail
#1 2 3 4 5 6 7 8 9
Tyr
Gapdh
B
sgRNA-Tyr-B+C+D+E Tail #5
GCGAAGGCACCGCCCTCTTTTGG//CCAGAAGCCAATGCACCTATCGG//CTTCATAACATCCAAGGATCTGG//TACAGCTACCTCCAAGAGTCAGG WT
CGAAGCCAt---------------//------------------------//-----------------ATCTGG//TACAGCTACCTCCAAGAGTCAGG -118 X6
GCC--------------------//------------------------//-----------------ATCTGG//TACAGCTACCTCCAAGAG----G -127 X3
CCAT-------------------//------------------------//-----------------ATCTGG//TACAGCTACCTCCAAGAGTCAGG -118 X1
GCGAAGGCA--------------//------------------------//------------------TCTGG//TACAGCTACCTCCAAG--TCAGG -122 X1
C
Blastocysts
sgRNA-Tyr-C
sgRNA-Tyr-B+C+D+E
#1 2 3 4 5 6 7 8
Tyr
D
sgRNA-Tyr-C Blastocyst #1
AGTTTACCCAGAAGCCAATGCACCTATCGGCCATAAC
AGTTTACCCAGAAGCCAATGCAC-TATCGGCCATAAC -1 X3
AGTTTACCCAGAAGCCAATGCACCTATCGGCCATAAC WT X2
AGTTTACCCAGAAGCCAATGC----------CATAAC -10 X3
GTTTACCCAGAAGCCAATGCACCcTATCGGCCATAAC +1 X3
E
sgRNA-Tyr-B+C+D+E Blastocyst #5
GCGAAGGCACCGCCCTCTTTTGG//CCAGAAGCCAATGCACCTATCGG//CTTCATAACATCCAAGGATCTGG//TACAGCTACCTCCAAGAGTCAGG
CGAAGGCACCGCCCA-------G//CCAGAAGCCAATGCAC-------//-----------------------//----------------------- -220 X6
CGAAGA-----------------//--AGAAGCCAATGCACC------//-----------------------//-----------------GTCAGG -137 X1
GCGAAGG----------------//------------------------//-----------------------//----------------------- -158 X2
F
Blastomeres
sgRNA-Tyr-C
sgRNA-Tyr-B+C+D+E
#1 2 3 4 5 6 7 8
#6
#8
G
16-cell embryo #4 (sgRNA-Tyr-C)
GCCCTCTTTTGGAAGTTTACCCAGAAGCCAATGCACCTATCGGCCATAACAGAGACTCTTACA
Blastomere
#1 GCCCTCTTTTGGAAGTTTACCCAGAAGCCAATGCAC--ATCGGCCATAACAGAGACTCTTACA -2
2 GCCCTCTTTTGGAAGTTTACCCAGAAGCCAATGCAC--ATCGGCCATAACAGAGACTCTTACA -2
3 GCCCTCTTTTGGAAGTTTACCCAGAAGCCAATGCAC-TATCGGCCATAACAGAGACTCTTACA -1
4 GCCCTCTTTTGGAAGTTTACCCAGAAGCCAATGCACCTATCGGCCATAACAGAGACTCTTACA WT
--- -149
5 GCCCTCTTTTGGAAGTTTACCCAGAAGCCAATGCAC--------------------TCTTACA -20
6 GCCCTCTTTTGGAAGTTTACCCAGAAGCCAATGCACCTATCGGCCATAACAGAGACTCTTACA WT
7 GCCCTCTTTTGGAAGTTTACCCAGAAGCCAATGCAC--ATCGGCCATAACAGAGACTCTTACA -2
8 GCCCTCTTTTGGAAGTTTACCCAGAAGCCAATGCACCTATCGGCCATAACAGAGACTCTTACA WT
H
16-cell embryo #6 (sgRNA-Tyr-B+C+D+E)
GCGAAGGCACCGCCCTCTTTTGG//CCAGAAGCCAATGCACCTATCGG//CTTCATAACATCCAAGGATCTGG//TACAGCTACCTCCAAGAGTCAGG
Blastomere
#1 G---------------------G//CCAGAAGCCAATGCACCTATCGG//CTTCATAACATCCAAG------G//TACAGCTACCTCCAAG-GTCAGG -28
2 GCGAAGGCACCGCCCTCT-----//------------------ATCGG//CTTCATAACATCCAAtccGGATCTGG//TACAGCTAaCCTCCAAGAGT -27
GCGAAGGCACCGCCC--TTTTGG//CCAGAAGCCAAT-----------//-----------------------//----------------------- -376
3 GCGAAG-----------------//CCAGAAGCCAATGCACC------//-----------------------//TACAGCTACCTCCAAGAGTCAGG -56
4 GCGAAGGCACCGCCCTCT-----//------------------ATCGG//CTTCATAACATCCAAtccGGATCTGG//TACAGCTAaCCTCCAAGAGT -27
GCGAAGGCACCGCCC--TTTTGG//CCAGAAGCCAAT-----------//-----------------------//----------------------- -376
5 G---------------------G//CCAGAAGCCAATGCACCTATCGG//CTTCATAACATCCAAG------G//TACAGCTACCTCCAAG-GTCAGG -28
6 GCGAAGGCACCGCCCTCT-----//------------------ATCGG//CTTCATAACATCCAAtccGGATCTGG//TACAGCTAaCCTCCAAGAGT -27
7 GCGAAGGCACCGCCCTCT-----//------------------ATCGG//CTTCATAACATCCAAtccGGATCTGG//TACAGCTAaCCTCCAAGAGT -27
GCGAAGGCACCGCCC--TTTTGG//CCAGAAGCCAAT-----------//-----------------------//----------------------- -376
8 G---------------------G//CCAGAAGC---------TATCGG//CTTCATAACATCCAAG------G//TACAGCTACCTCCAAG-GTCAGG -38

Supplementary information, Figure S1. Genotyping analysis of gene-edited mice by C-CRISPR.

(A) PCR products from nine mice with sgRNA-*Tyr*-B+C+D+E targeting. PCR products of mice #1 to #6 were TA cloned and sequenced. PCR products of tail marked with * were sequenced shown in Figure S1B. NC, negative control.

(B) Representative sequences from mouse tail #5 with sgRNA-*Tyr*-B+C+D+E targeting. The sgRNA targeting sequences are labeled in green and PAMs are labeled in red; deleted nucleotides are indicated by hyphens. Dashed lines mark the region omitted for clarity.

(C) Representative PCR products from whole embryos at blastocyst stage with sgRNA-*Tyr*-C or sgRNA-*Tyr*-B+C+D+E targeting. PCR products of blastocysts marked with * were further sequenced and shown in Figure S1D and S1E.

(D & E) Representative sequences from Blastocyst #1 (D) with sgRNA-*Tyr*-C targeting and Blastocyst #5 (E) with sgRNA-*Tyr*-B+C+D+E targeting.

(F) Representative PCR products from single blastomeres of 8- to 16-cell embryos with sgRNA-*Tyr*-C or sgRNA-*Tyr*-B+C+D+E targeting.

(G & H) Representative sequences for individual blastomeres from a single 16-cell embryo with sgRNA-*Tyr*-C targeting (G) and sgRNA-*Tyr*-B+C+D+E targeting (H). About 50% blastomeres of each embryo were successfully amplified and sequenced. The sgRNA targeting sequences are labeled in green and the PAM sequences are labeled in red; deleted nucleotides are indicated by hyphens. Dashed lines mark the region omitted for clarity.
