# Supplementary material for: One-step generation of complete gene knockout mice and monkeys by CRISPR/Cas9-mediated gene editing with multiple sgRNAs
Source: Cell Res. 2017 Jun 6;27(7):933–45. doi: 10.1038/cr.2017.81 (PMC5518993; doi:10.1038/cr.2017.81)
Supplement: Supplementary information, Figure S2 — Genotyping analysis of genegene-edited edited edited monkey embryos and tissues by C-CRISPR. [file cr201781x2.pdf]

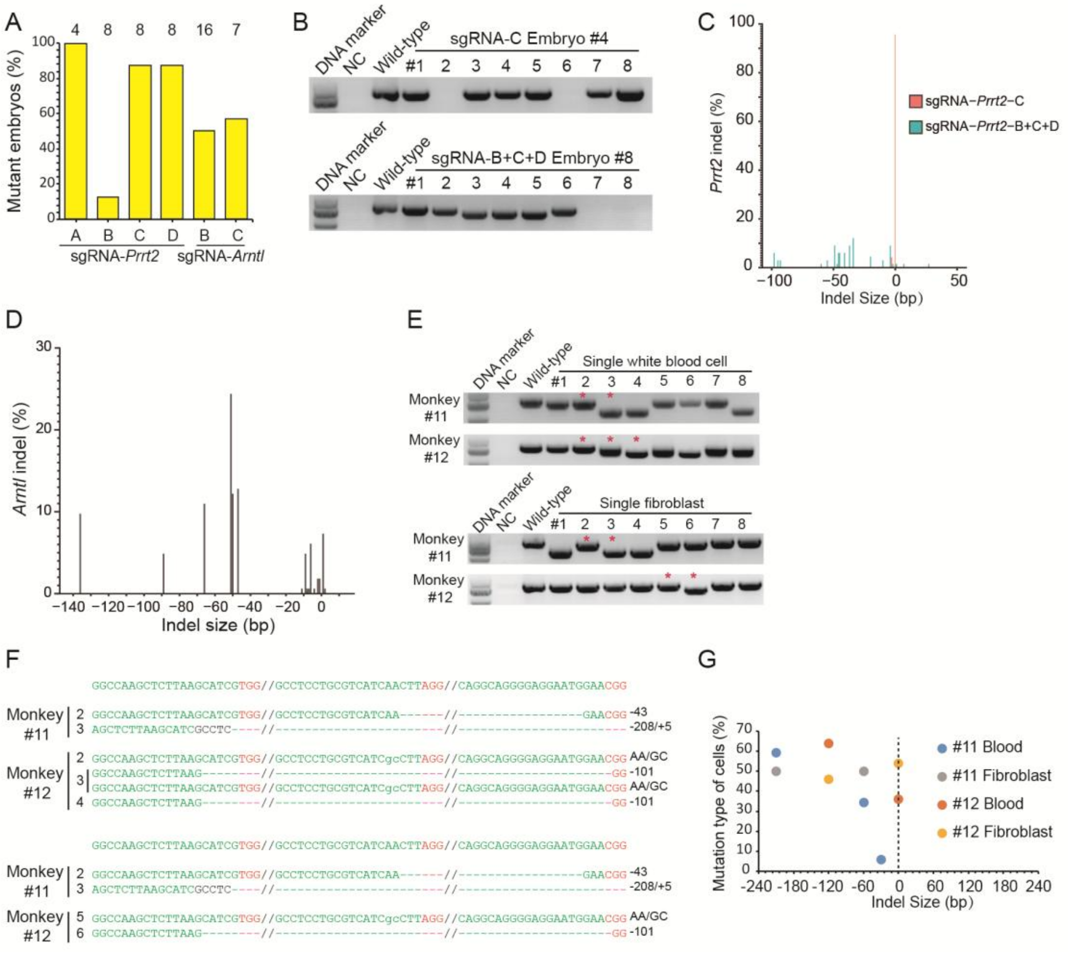
A
Mutant embryos (%)
4 8 8 8 16 7
A B C D B C
sgRNA-Prrt2
sgRNA-Arntl
B
DNA marker
NC
Wild-type
sgRNA-C Embryo #4
#1 2 3 4 5 6 7 8
sgRNA-B+C+D Embryo #8
C
Prrt2 indel (%)
sgRNA-Prrt2-C
sgRNA-Prrt2-B+C+D
Indel Size (bp)
D
Arntl indel (%)
Indel size (bp)
E
Single white blood cell
Single fibroblast
Monkey #11
Monkey #12
F
GGCCAAGCTCTTAAGCATCGTGG//GCCTCCTGCGTCATCAACTTAGG//CAGGCAGGGGAGGAATGGAACGG
Monkey #11
Monkey #12
-43
-208/+5
AA/GC
-101
G
Mutation type of cells (%)
#11 Blood
#11 Fibroblast
#12 Blood
#12 Fibroblast
Indel Size (bp)

Supplementary information, Figure S2. Genotyping analysis of gene-edited monkey embryos and tissues by C-CRISPR.

(A) Cleavage efficiency of each sgRNA used for *Prrt2* and *Arntl* targeting. Zygotes were injected with *Cas9* mRNA and single sgRNA, and then cultured into 8-cell embryos for genotyping. Number, total embryos used for genotyping.

(B) PCR products from blastomeres of embryo #4 with sgRNA-*Prrt2*-C and blastomeres of embryo #8 with sgRNA-*Prrt2*-B+C+D targeting. PCR products of blastomeres were TA cloned and sequenced.

(C & D) Size distribution of deletions or insertions in embryos with *Prrt2* and *Arntl* targeting. Data was from total alleles analyzed from blastomeres of 8-cell embryos with *Prrt2* targeting (C) and total TA clones sequenced from 8-cell embryos with *Arntl* targeting (D).

(E & F) Representative PCR products and sequences from single fibroblasts and blood cells of monkey #11 and #12. PCR products of the single cells marked with * were further sequenced and shown.

(G) Size distribution of deletions or insertions in blood cells and fibroblasts with *Prrt2* editing.
