# Supplementary material for: One-step generation of complete gene knockout mice and monkeys by CRISPR/Cas9-mediated gene editing with multiple sgRNAs
Source: Cell Res. 2017 Jun 6;27(7):933–45. doi: 10.1038/cr.2017.81 (PMC5518993; doi:10.1038/cr.2017.81)
Supplement: Supplementary information, Figure S3 — Off-target analysis of mice resulting from Tyr targeting and monkeys resulting from Prrt2 targeting. [file cr201781x3.pdf]

A

sgRNA-*Tyr*-B

locus	guide sequence	PAM	indel (n=6)
Target sequence	GCGAAGGCACCGCCCTCTTT	TGG	
chr17:57065718	GGGAAGGCAACACCCTCTTT	GGG	0/6
chr6:138140489	GCGCTGGCACCGCCCTTTTT	CAG	0/6
chr4:138750107	GTGCGGACACCGCCCTCTTT	TAG	0/6
chr2:32395585	TATAAGGCCCCGCCCTCTTT	CGG	0/6
chr9:23096850	GGGAAGGACTCGCCCTCTTT	CAG	0/6
chr17:24453551	TGGAGGGCACCACCCTCTTT	CAG	0/6
chr9:96832929	TGGAAGGTACCTCCCTCTTT	GAG	0/6
chr15:79169441	GCTCTGGCACCGCCCTGTTT	CAG	0/6
chr4:86233378	GCGAAGGCACCGCCCTCCTC	AGG	0/6
chr6:115975395	TGGAAGGCTCCTCCCTCTTT	CAG	0/6

sgRNA-*Tyr*-C

locus	guide sequence	PAM	indel (n=6)
Target sequence	CCAGAAGCCAATGCACCTAT	CGG	
chr8:73203311	CTAGAAGCCAATGCACCTAA	GGG	0/6
chr10:55107975	CCAGAATCCAATGCTCCTAT	TAG	0/6
chr10:116978236	CCGGAATCCAATGCACCTAC	AAG	0/6
chr10:124330321	CCCAAAGTAAATGCACCTAT	CAG	0/6
chr17:8532910	TTAGAAGACAATGCACCTAC	AAG	0/6
chr16:57712501	CCCAAAGCCCTTGCACCTAT	GAG	0/6
chr17:44031368	ATACAAGCCAATGCACATAT	GAG	0/6
chr3:6828323	ACAGAATTGAATGCACCTAT	AGG	0/6
chr11:28415232	ACAGAAAACATTGCACCTAT	CAG	0/6
chr1:33799882	TGAGCAGCCAATGCACCTTT	TGG	0/6

sgRNA-*Tyr*-D

locus	guide sequence	PAM	indel (n=6)
Target sequence	CTTCATAACATCCAAGGATC	TGG	
chr15:74536351	CTGCCTAGCATCCAAGGATC	AGG	0/6
chr18:43788370	CTTTTCAACATCCAAGGATC	CAG	0/6
chr15:61581619	CTTCCAAACCTCCAAGGATC	AGG	0/6
chr13:39330053	GCACATAACGTCCAAGGATC	AGG	0/6
chr14:80206603	CTTCTTGAAATCCAAGGATC	CGG	0/6
chr5:36335565	CCTCATAAAATCCAAGGATG	CAG	0/6
chr8:85681387	CTTAATAACACCCAAGGATA	TGG	0/6
chr3:98944708	CATCATAAAATCCAAGCATC	AAG	0/6
chr6:42584574	TTGCTTAACATGCAAGGATC	AAG	0/6
chr10:18410911	CTTCCTATCCACCAAGGATC	CAG	0/6

sgRNA-*Tyr*-E

locus	guide sequence	PAM	indel (n=6)
Target sequence	TACAGCTACCTCCAAGAGTC	AGG	
chr8:108702504	TACAGCTACCTCCAAGAGTT	TGG	0/6
chrX:130677312	CACAGCTACCTCTAAGAGTC	TAG	0/6
chr3:130295069	AACAGCTTCCACCAAGAGTC	TGG	0/6
chr3:133082201	GGCTGCTCCCTCCAAGAGTC	TGG	0/6
chrX:147311924	TGATGCTGCCTCCAAGAGTC	AGG	0/6
chr1:168151597	TATAGCTGCCTCAAAGAGTC	AGG	0/6
chr5:50071052	TTCAGCAGCTTCCAAGAGTC	AAG	0/6
chr2:7777791	GACAGCTATCTTCAAGAGTC	TGG	0/6
chr10:84425609	TACCCCTGCCACCAAGAGTC	TGG	0/6

B

sgRNA-*Prrt2*-B

locus	guide sequence	PAM	indel (n=2)
Target sequence	GGCCAAGCTCTTAAGCATCG	TGG	
chr8:8862302	GTTTAAGCAGTTAAGCATCG	TGG	0/2
chr7:142439417	GGCCTCCCTGTTAAGCATCG	GGG	0/2
chr16:18400530	GGCCTCCCTGTTAAGCATCG	GGG	0/2
chr3:1322473581	TGCCAAGCTCTTAACCATCA	TGG	0/2
chr17:9980144	TGGCCAGATCTTAAGCATCC	AGG	0/2
chr10:63847987	TGAGAAGCTGTTAAGCATCA	CGG	0/2
chr8:42993888	GGAAAAGATATTAAGCATCA	AGG	0/2

sgRNA-*Prrt2*-C

locus	guide sequence	PAM	indel (n=2)
Target sequence	GCCTCCTGCGTCATCAACTT	AGG	
chr5:72228276	TCTCCCTGCTTCATCAACTT	AGG	0/2
chr1:190035638	AAGGCCTGCTTCATCAACTT	GGG	0/2
chr9:69194244	GGCACCTGCAGCATCAACTT	TGG	0/2
chr15:40107119	GCTTAATGCATCATCAACTT	GGG	0/2
chr20:61058621	GCCTCCAGCATCATCACCTT	GGG	0/2
chr10:85811043	GAGTCCTTCGTCATCAACCT	GGG	0/2

sgRNA-*Prrt2*-D

locus	guide sequence	PAM	indel (n=2)
Target sequence	CAGGCAGGGGAGGAATGGAA	CGG	
chr3:88667957	AAGCCAGTGGAGGAATGGAA	GGG	0/2
chr7:96903885	GAGGGAGGGAAGGAATGGAA	GGG	0/2
chr3:10010150	GAGGGAGGGAAGGAATGGAA	TGG	0/2
chr2:37733632	GAGGGAGGTGAGGAATGGAA	CGG	0/2
chr12:127960318	CAGACAGAGGAGGAATGGAT	GGG	0/2

C

sgRNA-*Prrt2*-C	GCCTCCTGCGTCATCAACTTAGG	
v5.0	CCCTCCTGCTTCAGCCTCTTGAG	
WGS #11	---TCCTGCTTCAGCCTCTTGAG	8/27 reads
#8	CTCTCCTGCTTCAGCCTCTTGAG<br>C	
#9	CTCTCCTGCTTCAGCCTCTTGAG	
#11	CTCTCCTGCTTCAGCCTCTTGAG<br>TG	
#12	CTCTCCTGCTTCAGCCTCTTGAG<br>C	

D

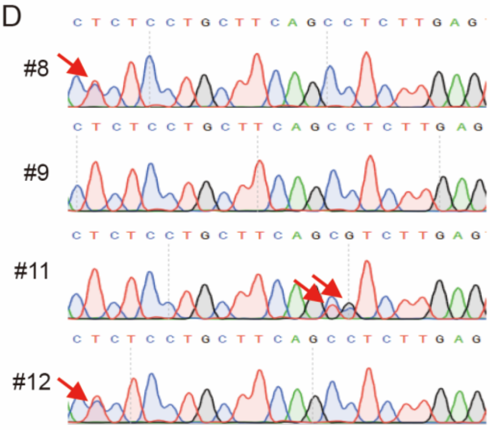

Supplementary information, Figure S3. Off-target analysis of mice resulting from *Tyr* targeting and monkeys resulting from *Prrt2* targeting.

(A) Six mice from sgRNA-*Tyr*-B+C+D+E targeting were used for off-target analysis. Up to 10 potential off-target sites for each sgRNA were selected for analysis. DNA sequencing of PCR products amplified from these genomic sites were TA cloned and sequenced. Red, mismatch with targeted sequence.

(B) Two live monkeys with multiple sgRNA-*Prrt2*-A+B+C targeting (#11, #12) were used for off-target analysis. The off-target sites with up to three mismatches for each sgRNA were selected for analysis. Red, mismatch with targeted sequence.

(C&D) Sequence analysis of the off-target site in monkey #11. DNA samples from monkey #8, #9, #11 and #12 were amplified and sequenced. Red arrow, bimodal sequence.
