# Supplementary material for: One-step generation of complete gene knockout mice and monkeys by CRISPR/Cas9-mediated gene editing with multiple sgRNAs
Source: Cell Res. 2017 Jun 6;27(7):933–45. doi: 10.1038/cr.2017.81 (PMC5518993; doi:10.1038/cr.2017.81)
Supplement: Supplementary information, Table S1 — Phenotypic analysis of F0 mice with Y-chromosome gene deletions generated by C-CRISPR [file cr201781x4.pdf]

Supplementary information, Table S1. Phenotypic analysis of F0 mice with Y-chromosome gene deletions generated by C-CRISPR

Gene	Sample	Concentration (M/mL)	Testis/body weight (g)	Motile Sperm (%)	Progressive Sperm (%)	Reproduction	Genotype
WT	#1	9.1	0.403	76	64		WT
WT	#1	15.4	0.376	74	59		
WT	#2	22.5	0.360	80	67		WT
WT	#2	9.2	0.339	70	61		
Eif2s3y	#1	0.5	0.061	0.0	0.0		Pure KO
Eif2s3y	#1	0.4	0.057	0.0	0.0		
Eif2s3y	#2	2.3	0.053	0.0	0.0		Pure KO
Eif2s3y	#2	3.3	0.052	0.0	0.0		
Eif2s3y	#3	2.7	0.052	0.0	0.0		Pure KO
Eif2s3y	#3	1.9	0.048	0.0	0.0		
Eif2s3y	#4					Infertilty	Pure KO
Eif2s3y	#4						
Eif2s3y	#5					Infertilty	Pure KO
Eif2s3y	#5						
Eif2s3y	#6					Infertilty	Pure KO
Eif2s3y	#6						
Zfy1	#1	20.6	0.318	70	62		Pure KO
Zfy1	#1	8.2	0.269	68.0	55.0		
Zfy1	#2	26.5	0.322	78	64		Pure KO
Zfy1	#2	14.0	0.333	76	64		
Zfy1	#3	14.7	0.333	75	63		Pure KO
Zfy1	#3	19.5	0.309	73	61		
Zfy1	#4					Fertility	Pure KO
Zfy1	#4						
Zfy1	#5					Fertility	Pure KO
Zfy1	#5						
Zfy1	#6					Fertility	Pure KO
Zfy1	#6						
Ube1y1	#1	6.6	0.338	64	50		Pure KO
Ube1y1	#1	22	0.349	74	59		
Ube1y1	#2	23.1	0.311	70	57		Pure KO
Ube1y1	#2	23.1	0.330	61	48		
Ube1y1	#3					Fertility	Pure KO
Ube1y1	#3						
Ube1y1	#4					Fertility	Pure KO
Ube1y1	#4						
Ube1y1	#5					Fertility	Pure KO
Ube1y1	#5						

Kdm5d	#1		0.230				Pure KO
Kdm5d	#1		0.264				
Kdm5d	#2		0.231				Pure KO
Kdm5d	#2		0.246				
Kdm5d	#3	14.7	0.292	73.0	60.0		Pure KO
Kdm5d	#3	14.7	0.306	75.0	62.0		
Kdm5d	#4	19.9	0.280	69.0	55.0		Pure KO
Kdm5d	#4	9.2	0.321	61.0	48.0		
Kdm5d	#5	24.3	0.329	72.0	58.0		Pure KO
Kdm5d	#5	10.8	0.335	74.0	61.0		
Kdm5d	#6	8.5	0.276	73.0	63.0		Pure KO
Kdm5d	#6	19.3	0.253	68.0	55.0		
Kdm5d	#7	4.1	0.220	77.0	69.0		Pure KO
Kdm5d	#7	1.6	0.223	55.0	48.0		
Kdm5d	#8	13.3	0.255	75.0	58.0		Pure KO
Kdm5d	#8	6.4	0.241	75.0	63.0		
Kdm5d	#9					Fertility	Pure KO
Kdm5d	#9						
Kdm5d	#10					Fertility	Pure KO
Kdm5d	#10						
Kdm5d	#11					Fertility	Pure KO
Kdm5d	#11						
Ddx3y	#1	14.4	0.294	73.0	61.0		Pure KO
Ddx3y	#1	13.3	0.293	75.0	62.0		
Ddx3y	#2	4.1	0.224	56.0	44.0		Pure KO
Ddx3y	#2	7	0.214	58.0	48.0		
Ddx3y	#3	0.3	0.056	0	0		Pure KO
Ddx3y	#3	2	0.054	0	0		
Ddx3y	#4	4.8	0.244	80	71		Pure KO
Ddx3y	#4	10.6	0.236	74	65		
Ddx3y	#5	9	0.252	81	64		Pure KO
Ddx3y	#5	8.9	0.227	77	62		
Ddx3y	#6					Fertility	Pure KO
Ddx3y	#6						
Ddx3y	#7					Fertility	Pure KO
Ddx3y	#7						
Ddx3y	#8					Fertility	Pure KO
Ddx3y	#8						
Usp9y	#1	18.3	0.323	70.0	56.0		Pure KO
Usp9y	#1	16.4	0.306	72.0	59.0		
Usp9y	#2	5.8	0.361	72.0	61.0		Pure KO
Usp9y	#2	24.1	0.358	76.0	60.0		

Usp9y	#3	22.1	0.343	72.0	60.0		Pure KO
Usp9y	#3	14	0.347	69.0	57.0		
Usp9y	#4	24	0.328	73.0	59.0		Pure KO
Usp9y	#4	17.2	0.354	73.0	60.0		
Usp9y	#5					Fertility	Pure KO
Usp9y	#5						
Usp9y	#6					Fertility	Pure KO
Usp9y	#6						
Usp9y	#7					Fertility	Pure KO
Usp9y	#7						

WT, wild type.

Pure KO, mice with complete knockout mutations.

Related to Figure 2.
