# Supplementary material for: One-step generation of complete gene knockout mice and monkeys by CRISPR/Cas9-mediated gene editing with multiple sgRNAs
Source: Cell Res. 2017 Jun 6;27(7):933–45. doi: 10.1038/cr.2017.81 (PMC5518993; doi:10.1038/cr.2017.81)
Supplement: Supplementary information, Table S2 — Summary of HiSeq X Ten sequencing [file cr201781x5.pdf]

Supplementary information, Table S2. Summary of HiSeq X Ten sequencing

**Sample**	**Species**	**Treatment**	**Accession**	**Mapped bases (Gbp)**	**Coverage**
Prrt2-#8	Monkey	SgRNA-*Prrt2*-A	SRR5273327	56.80	19.54
Prrt2-#9	Monkey	SgRNA-*Prrt2*-A	SRR5273326	59.46	20.45
Prrt2-#11	Monkey	SgRNA-*Prrt2*-B+C+D	SRR5004143	72.89	25.07
Prrt2-#12	Monkey	SgRNA-*Prrt2*-B+C+D	SRR5004146	67.90	23.35
Tyr-#1	Mouse	SgRNA-*Tyr*-B+C+D+E	SRR5004147	45.43	16.31
Tyr-#2	Mouse	SgRNA-*Tyr*-B+C+D+E	SRR5004145	47.94	17.21
Tyr-#3	Mouse	SgRNA-*Tyr*-B+C+D+E	SRR5004148	45.90	16.48
Tyr-#4	Mouse	SgRNA-*Tyr*-B+C+D+E	SRR5004149	49.93	17.93
Tyr-#5	Mouse	SgRNA-*Tyr*-B+C+D+E	SRR5004142	45.97	16.50
Tyr-#6	Mouse	SgRNA-*Tyr*-B+C+D+E	SRR5004144	46.85	16.82
