# Supplementary material for: One-step generation of complete gene knockout mice and monkeys by CRISPR/Cas9-mediated gene editing with multiple sgRNAs
Source: Cell Res. 2017 Jun 6;27(7):933–45. doi: 10.1038/cr.2017.81 (PMC5518993; doi:10.1038/cr.2017.81)
Supplement: Supplementary information, Table S4 — sgRNA sequences and primers sgRNA sequences [file cr201781x7.pdf]

Supplementary information, Table S4. sgRNA sequences and primers

sgRNA sequences

Locus	Sequence (5'-3')	Figure
GFP-A	AAGGGCGAGGAGCTGTTCACCGG	1
GFP-B	CTGAAGTTCATCTGCACCACCGG	1
GFP-C	GGAGCGCACCATCTTCTTCAAGG	1
GFP-D	GGTGAACCGCATCGAGCTGAAGG	1
Tyr-A	GGGAAGGGTTACTCAGAGTCAGG	2
Tyr-B	GCGAAGGCACCGCCCTCTTTTGG	2
Tyr-C	CCAGAAGCCAATGCACCTATCGG	2
Tyr-D	CTTCATAACATCCAAGGATCTGG	2
Tyr-E	TACAGCTACCTCCAAGAGTCAGG	2
Tyr-F	CAATGTGGGTAACCTCTTTGGGG	2
Tet1-A	GGCATGCTGGACTTCATTCTCGG	3
Tet1-B	GATGTCCATGCCGGTTACACAGG	3
Tet1-C	GAAGCCAGAGGCCACCTCACAGG	3
Tet2-A	CAGGGAGCAAGAGATTCCGAAGG	3
Tet2-B	TCAGTCCTCCACTCTCAGACCGG	3
Tet2-C	GTGAACCAAGGACCGTCTCCAGG	3
Tet3-A	CCCTACTTCCACAGAGCCTCAGG	3
Tet3-B	GCCTGTTAGGCAGATTGTTCTGG	3
Tet3-C	ACAAGCTGGAGGAGCTCATCCGG	3
Zfy1-A	GGATAGTGACCAGATTGTTGTGG	4
Zfy1-B	CCTGAGCAAGTTCTCAATTTAGG	4
Ubey1y-A	GAAACCCCTACTTGCCTCGCTGG	4
Ubey1y-B	CTCAGCACAGCAGGCCTCCTCGG	4
Kdm5d-A	GATGGTACCTACAGAAGTTGTGG	4
Kdm5d-B	GGACTTATCTCCTGAAGAAAAGG	4
Eif2s3y-A	ACAATTGGTCATGTTGCTCATGG	4
Eif2s3y-B	AGGAATATTACCATAAAACTTGG	4
Ddx3y-A	GTCTGTGATAAGGACAGTTCAGG	4
Ddx3y-B	GTGATCGTGGAAGTGGATCCAGG	4
Usp9y-A	ACTCTGGCTCTGTGTTTCCCAGG	4
Usp9y-B	AGGATTAGACTACCTTTGGAAGG	4
Sry-A	GCATTTATGGTGTGGTCCCGTGG	4
Sry-B	CGAAAAAAGGCCCTTTTTCCAGG	4
Erdr1-A	ACGGACGGACTCCACAAGGTAGG	4
Erdr1-B	TGGCACATACATCTTGACCGCGG	4
Prrt2-A	GATGGCAGCCAGCAGCTCTGAGG	5
Prrt2-B	GGCCAAGCTCTTAAGCATCGTGG	5
Prrt2-C	GCCTCCTGCGTCATCAACTTAGG	5
Prrt2-D	CAGGCAGGGGAGGAATGGAACGG	5

ArntL-A	GCTGGCCACCCACAAAGATGGGG	5
ArntL-B	GCAGTCGTCCAATTGCAACGAGG	5
ArntL-C	GTTTCTCGGCACGCGATAGATGG	5

Primer sequences used to generate the templates for sgRNA *in vitro* transcriptions.

Locus	Sequence (5'-3')	Figure
GFP-A	TAATACGACTCACTATAGGGAAGGGCGAGGAGCTGTTCACGTTTTAGAGCTAGAAATAG	1
GFP-B	TAATACGACTCACTATAGGGCTGAAGTTCATCTGCACCACGTTTTAGAGCTAGAAATAG	1
GFP-C	TAATACGACTCACTATAGGGGGAGCGCACCATCTTCTTCAGTTTTAGAGCTAGAAATAG	1
GFP-D	TAATACGACTCACTATAGGGGGTGAACCGCATCGAGCTGAGTTTTAGAGCTAGAAATAG	1
Tyr-A	TAATACGACTCACTATAGGGGGGAAGGGTTACTCAGAGTCGTTTTAGAGCTAGAAATAG	2
Tyr-B	TAATACGACTCACTATAGGGGCGAAGGCACCGCCCTCTTTGTTTTAGAGCTAGAAATAG	2
Tyr-C	TAATACGACTCACTATAGGGCCAGAAGCCAATGCACCTATGTTTTAGAGCTAGAAATAG	2
Tyr-D	TAATACGACTCACTATAGGGCTTCATAACATCCAAGGATCGTTTTAGAGCTAGAAATAG	2
Tyr-E	TAATACGACTCACTATAGGGTACAGCTACCTCCAAGAGTCGTTTTAGAGCTAGAAATAG	2
Tyr-F	TAATACGACTCACTATAGGGCAATGTGGGTAACCTCTTTGGTTTTAGAGCTAGAAATAG	2
Tet1-A	TAATACGACTCACTATAGGGGGCATGCTGGACTTCATTCTGTTTTAGAGCTAGAAATAG	3
Tet1-B	TAATACGACTCACTATAGGGGATGTCCATGCCGGTTACACGTTTTAGAGCTAGAAATAG	3
Tet1-C	TAATACGACTCACTATAGGGGAAGCCAGAGGCCACCTCACGTTTTAGAGCTAGAAATAG	3
Tet2-A	TAATACGACTCACTATAGGGCAGGGAGCAAGAGATTCCGAGTTTTAGAGCTAGAAATAG	3
Tet2-B	TAATACGACTCACTATAGGGTCAGTCCTCCACTCTCAGACGTTTTAGAGCTAGAAATAG	3
Tet2-C	TAATACGACTCACTATAGGGGTGAACCAAGGACCGTCTCCGTTTTAGAGCTAGAAATAG	3
Tet3-A	TAATACGACTCACTATAGGGCCCTACTTCCACAGAGCCTCGTTTTAGAGCTAGAAATAG	3
Tet3-B	TAATACGACTCACTATAGGGGCCTGTTAGGCAGATTGTTCGTTTTAGAGCTAGAAATAG	3
Tet3-C	TAATACGACTCACTATAGGGACAAGCTGGAGGAGCTCATCGTTTTAGAGCTAGAAATAG	3
Zfy1-A	TAATACGACTCACTATAGGGGGATAGTGACCAGATTGTTGGTTTTAGAGCTAGAAATAG	4
Zfy1-B	TAATACGACTCACTATAGGGCCTGAGCAAGTTCTCAATTTGTTTTAGAGCTAGAAATAG	4
Ube1y1-A	TAATACGACTCACTATAGGGGAAACCCCTACTTGCCTCGCGTTTTAGAGCTAGAAATAG	4
Ubey1y-B	TAATACGACTCACTATAGGGCTCAGCACAGCAGGCCTCCTGTTTTAGAGCTAGAAATAG	4
Kdm5d-A	TAATACGACTCACTATAGGGGATGGTACCTACAGAAGTTGGTTTTAGAGCTAGAAATAG	4
Kdm5d-B	TAATACGACTCACTATAGGGGGACTTATCTCCTGAAGAAAGTTTTAGAGCTAGAAATAG	4
Eif2s3y-A	TAATACGACTCACTATAGGGACAATTGGTCATGTTGCTCAGTTTTAGAGCTAGAAATAG	4
Eif2s3y-B	TAATACGACTCACTATAGGGAGGAATATTACCATAAAACTGTTTTAGAGCTAGAAATAG	4
Ddx3y-A	TAATACGACTCACTATAGGGGTCTGTGATAAGGACAGTTCGTTTTAGAGCTAGAAATAG	4
Ddx3y-B	TAATACGACTCACTATAGGGGTGATCGTGGAAGTGGATCCGTTTTAGAGCTAGAAATAG	4
Usp9y-A	TAATACGACTCACTATAGGGACTCTGGCTCTGTGTTTCCCGTTTTAGAGCTAGAAATAG	4
Usp9y-B	TAATACGACTCACTATAGGGAGGATTAGACTACCTTTGGAGTTTTAGAGCTAGAAATAG	4
Sry-A	TAATACGACTCACTATAGGGGCATTTATGGTGTGGTCCCGGTTTTAGAGCTAGAAATAG	4
Sry-B	TAATACGACTCACTATAGGGCGAAAAAAGGCCCTTTTTCCGTTTTAGAGCTAGAAATAG	4
Erdr1-A	TAATACGACTCACTATAGGGACGGACGGACTCCACAAGGTGTTTTAGAGCTAGAAATAG	4
Erdr1-B	TAATACGACTCACTATAGGGTGGCACATACATCTTGACCGGTTTTAGAGCTAGAAATAG	4
Prrt2-A	GAAATTAATACGACTCACTATAGGGCTGCAGCAGCTGGTTGAGGGTTTTAGAGCTAGAAATAGC	5

Prrt2-B	GAAATTAATACGACTCACTATAGGGGCCAAGCTCTTAAGCATCGGTTTTAGAGCTAGAAATAGC	5
Prrt2-C	GAAATTAATACGACTCACTATAGGGCCTCCTGCGTCATCAACTTGTTTTAGAGCTAGAAATAGC	5
Prrt2-D	GAAATTAATACGACTCACTATAGGCAGGCAGGGGAGGAATGGAAGTTTTAGAGCTAGAAATAGC	5
ArntL-A	TAATACGACTCACTATAGGGGCTGGCCACCCACAAAGATGGTTTTAGAGCTAGAAATAG	5
ArntL-B	TAATACGACTCACTATAGGGGCAGTCGTCCAATTGCAACGGTTTTAGAGCTAGAAATAG	5
ArntL-C	TAATACGACTCACTATAGGGGTTTCTCGGCACGCGATAGAGTTTTAGAGCTAGAAATAG	5
sgRNA-R	AAAAGCACCGACTCGGTGCC	

Cas9 mRNA	F	TAATACGACTCACTATAGGGAGATTTCAGGTTGGACCGGTG
	R	GACGTCAGCGTTCGAATTGC

Primers used for genotyping.

Locus	Sequence (5'-3')	Product (bp)	Figure
Tyr	Outer F: GTTATCCTCACACTACTTCTG	807	2
	Outer R: GTAATCCTACCAAGAGTCTCA		
	Inner F: TCCTCACACTACTTCTGATG	788	2
	Inner R: GTCTCAAGATGGAAGATCAC		
Eif2s3y	Outer F: ATCACAGGAATCTGGAAGC	834	4
	Outer R: CTACTAAGCAACTGTCACATC		
	Inner F: CAGGCTCACAGAGGTCTA	514	4
	Inner R: ACTCATCAGGTGTACTACTTC		
Zfy1	Outer F: CACAATAAGAAATATATGC	799	4
	Outer R: CAGATAATCCTCACAGTTGATTTTGT		
	Inner F: GTTACATAACCTAAAAGTAGAC	575	4
	Inner R: TATAGGATCAGTGATGACTTCTG		
Ube1y1	Outer F: GCAAAGGCACAGATTAGAC	769	4
	Outer R: GGTTGTCAGTAGCAGGAG		
	Inner F: TGGAGGCATTGTCAGTCA	625	4
	Inner R: ATGGCATCAGAAGCACAC		
Kdm5d	Outer F: GCAGGCTACACAGGAGTA	491	4
	Outer R: AGGGACAGTAACAGGCATA		
	Inner F: TTGGTGAGATGGCTGACT	457	4
	Inner R: GGACAGTAACAGGCATATGA		
Ddx3y	Outer F: CACTATACTACCAAGCCACAT	684	4
	Outer R: CCAAGACATCCAATCTCATAAG		
	Inner F: TTCTCACAATGTAGCTCAGT	512	4
	Inner R: AGGTAAGTAGGTAGACAGATTC		
Usp9y	Outer F: AGATGTAAGTTCAGCTAGGT	875	4
	Outer R: CACACTTTCTACTAACAGTCAC		
	Inner F: GTTGGGTATGTCTTTAGGTT	702	4

	Inner R: CATGAAGGACAACAGGTAA		
Sry	Outer F: GCACATTTTGGTCAGTGGCT	632	4
	Outer R: GCTCTACTCCAGTCTTGCCT		
	Inner F: GTTCAGCCCTACAGCCACATG	355	4
	Inner R: GCAGGCTGTAAAATGCCACTC		
Prrt2-Exon3	Outer F: GGAGGGATATGGAAACACAT	937	5
	Outer R: GATGCCAAGACAATGGAAGT		
	Inner F: TTTCCACCTGATCCCTTCTG	776	5
	Inner R: GAGGTCCAGAGAATTTGGGA		
ArntL-Exon 13	Outer F: CCAGTACCTCCAATTTCACTTC	1117	5
	Outer R: CAGAGACTCACATCACTTGTC		
	Inner F: ATACATTCTAGTCCCTCAGC	950	5
	Inner R: GGTACAATCACAGCTTACTG		

Primers used for off-target analysis.

sgRNA	Locus	Sequence	Product (bp)
sgRNA-*Tyr*-B	chr17:57065718	F: CCATTTACCTCATAGGACCT	617
		R: GTTGCTGATCTGAAGAAGTG	
	chr6:138140489	F: GGTATTCACAGCAATAGAGC	777
		R: GCAGTGACTTCTCATCTTGT	
	chr4:138750107	F: TTCTCTATCTGAGGCATCTG	701
		R: GACTCTACTGTTGTAGGACC	
	chr2:32395585	F:AACTCCTGCCCTACTTAGAAG	748
		R:CAATGATGACCTGTAACTCGTG	
	chr9:23096850	F:AAGATGCCTCCTGTTCTGAAG	671
		R:CAATCCATCATGCCTCTGAAG	
	chr17:24453551	F:CTGTCCTGGAACTCACTCTAT	764
		R:GACATTGGTTCTCAGACTTCC	
	chr9:96832929	F:GCTAATGCTAAGCCCTAACTG	633
		R:CATTCTCTGTGTCTGACTCTG	
	chr15:79169441	F:GTATATGTGTATGCCTGCGTC	1075
		R:ACAGAGTGAGTTCTAGGACAG	
	chr4:86233378	F:AGTGCGATACCTTACTGTCTC	659
		R:CATGTAGCAGCCTAGTTCTTC	
	chr6:115975395	F:GATACTTCTGGTCTTCAGCAG	625
		R:CATCCACAGCAGATACTAAGG	
sgRNA-*Tyr*-C	chr8:73203311	F: GCCTGTAATGATTGGACTGT	760
		R: GGACCTAACTATTGTGGGAAG	
	chr10:55107975	F: CATAGACTGTAGGACCAAGAC	625
		R: ACCCTTACCACCTGTTTATG	
	chr10:116978236	F: AGGAGCTGCATCATCTTATG	680

		R: ACAATCTGGAAGGCGAATAG	
	chr10:124330321	F:CAACCTCAAGAGTCAGACAAG<br>R:CCAGCAGTGAATTTCCATCAG	731
	chr17:8532910	F:CTGTGTATTTGTGGGACCTATG<br>R:GATTAGTCCATCAGAAGCAGAG	748
	chr16:57712501	F:GTCCTCTACTATTCTGTGTCTG<br>R:GATTGGTCTCTGTATAGTTGGC	639
	chr17:44031368	F:CCAATGACATACTCAGTGCTC<br>R:AGAGATGAGAGTTGGAGACAG	743
	chr3:6828323	F:CTCTACTTCCACTGAGGGTAC<br>R:GGACAATTCTCTTGGTTCTCC	703
	chr11:28415232	F:TAACCTACCTTACCACCATCC<br>R:GCCTATTGACCAGAGTTAGTG	718
	chr1:33799882	F:TAGTGTCACCTAGTAGAGTCC<br>R:GAGTAGCCAGTATGCTTAACC	857
sgRNA-*Tyr*-D	chr15:74536351	F: AGACAGAGGAAGCATTGAG<br>R: CTCCAGGTCATACACTGAG	516
	chr18:43788370	F: CCTGGATATGGCAGTCTCTA<br>R: CTGATTCTCTGAGAGGTGGTA	957
	chr15:61581619	F: GTGAAGGACTCAGGTTACTA<br>R: ATGATACAGTGGTAGCAGAC	799
	chr13:39330053	F:CATGGCTCAGGTACATGCTATGA<br>R:GGGCTTCCGATGGTGCGTGTC	998
	chr14:80206603	F:GTGGGCACAGTTGCTGATGAT<br>R:GCAGTTCCAACCCAATGCTCAC	752
	chr5:36335565	F:ATGAATCAGACCTTGCCCGTCAC<br>R:CATGCTGGGTGAAGGCAAGATC	653
	chr8:85681387	F:TGGACCATTAGACAGTGGGAGA<br>R:AGGCAGAAGAGGGTGAGTGGT	941
	chr3:98944708	F:TACCTTGGGTCCTTATGGTTGTT<br>R:TTGGCAGTAGTTGATGAGGAGAAGT	766
	chr6:42584574	F:TCTGTAAGGACACGAGTTAGGGATC<br>R:TTTGCTCCTATGACCTGGGTTCC	893
	chr10:18410911	F:GTACTACTTGAATTAGCAGCCACTGC<br>R:AAGTAGCCATGCAAAGCCAAGC	694
sgRNA-*Tyr*-E	chr8:108702504	F: GCTTTGATGTACTGGGAATC<br>R: GCTTAGAATGGAGGTCACTT	757
	chrX:130677312	F: GACAGAAGGATGAGGAATGA<br>R: CCAACCACTATTGAACTCAC	700
	chr3:130295069	F: CTTGTCATAGCAGTGTCCTG<br>R: GCTCCTTGAGAGTCCATTAG	643
	chr7:123982528	F:TAGCCCGCAGTGCCTCCCACAG<br>R:CTAGTCGCGGCGGCACCTGCTC	766

	chr3:133082201	F:CCTGCATCCAGCTAAATGGCACA<br>R:AGGCTGCCATACCTCCAACAA	1045
	chrX:147311924	F:CAGCCAAGGTGGGCACAAGGT<br>R:CAGGCATCCTCACAGGAAATTATGG	830
	chr1:168151597	F:TGAGCATGTCTGACTTCCCAGTT<br>R:AAGGTTACATTATGCCTCAGTTGT	745
	chr5:50071052	F:CAGGGACAGCATCCAGGTTTAT<br>R:GTGCCATCAATCACTAAGGCAAATC	782
	chr2:7777791	F:AAGCTGAAAGTGACTGGGAGA<br>R:CTGGGTGTATTTGTGCCTGTGT	973
	chr10:84425609	F:CATTTGTCAAACAGGAGCCACG<br>R:TGGTAATGAGGTAGGGACAGGC	807
sgRNA-*Prrt2*-B	chr16:19639092	F:GGTAGGTATTGCCAGATCCAA<br>R:CGGGTTCAAGTGATTCTTCTG	631
	chr8:8862302	F:GTTGAGGATCACTGTTCTACC<br>R:CTTGTCCTGTTTGTAGGTTGG	763
	chr7:142439417	F:ACAGACCTACAGAAGTGACTC<br>R:GCACGGAATCTATACAGAGAC	677
	chr16:18400530	F:ACAGACCTACAGAAGTGACTC<br>R:CAGAGACAGGCTAAGATATTGC	663
	chr3:1322473581	F:GAGTGTCTTGTATGGGCTAGA<br>R:CTCTGTGATTCCTGGTATCTG	967
	chr7:9980144	F:ACAAGTCTGGAGATTCCTTC<br>R:GATGTTCCTTGGTGTGATTC	937
	chr10:63847987	F:CCCACCATCAACCTAGATTTG<br>R:GTCCTCACAGAACTCTCATTC	706
	chr8:42993888	F:CCAGAGCAGTTACTTCAGAAG<br>R:GGCTAGTGGTTACAGTATTGG	944
	chr13:43492573	F:GCACAGACCTAGCTAATCTCG<br>R:CTTCCTTCCTTCCTTCCTTCC	806
	chr14:3502008	F:GGACATGGATGAAGTTGGAAG<br>R:TCAGTAATCTACAGAGGAGGTG	696
sgRNA-*Prrt2*-C	chr20:61736084	F:ATGACCTAATCCATGCTCCAG<br>R:GAACTCCTGACCTCAGATGAT	890
	chr5:72228276	F:CAATTCAGTGGGAAGTATAGGG<br>R:GACACAGTTGTTCACCATCTAC	882
	chr7:83976766	F:CCTAAACTCCCTTGACTTCTG<br>R:CCTGTTGTAACTCACTTCCTG	958
	chr1:190035638	F:GATACAGACTCAGAGGAATGG<br>R:CAGGAGGTCTTCATCTTCAAC	703
	chr9:69194244	F:GGTAGGCAAGTATGGTAAAGG<br>R:GGATTGTATCCTGGTGACTTC	605
	chr3:19342682	F:GATCACCTGAGGTTAGGAGTT	708

		R:CAGATTCTAGCTGAGTGGATG	
	chr15:40107119	F:CCTCATCATCTTCATGTCGAG<br>R:CATCTACTCACTTCCTCTAGC	792
	chr20:61058621	F:GGGAACTGAATGAACTGATGG<br>R:CCTGAAGTGAGGCAATAAGAG	700
	chr14:112810197	F:GAGAACAGTGTCCTTGATGAG<br>R:GGAGGTAGAGGTTGTAGTGAG	897
	chr10:85811043	F:CAACAGACAAGGCTAACTCAG<br>R:GACAGAGTGAGACTCCATTAC	637
sgRNA-*Prrt2*-D	chr3:88667957	F:GGTAAGTCTGGAACCAATAGG<br>R:CAGTCAAGCAGTATCTCTGAG	635
	chr7:96903885	F:CTCCTTACATCTCATTGGTCAG<br>R:CTATCCATCCTTCGTGATCTG	970
	chr3:10010150	F:CTCATCCTGTTCTACACCAAG<br>R:GAGTAAGGGAATCCATCTCTC	665
	chr2:37733632	F:CCTACTATACACAAGGTACTGG<br>R:CTAGAGGAAGACTGCTAAGTG	794
	chr12:127960318	F:GCTCACCATATCCAACTCATAC<br>R:CTCCATCCACTCTCCATCTAT	905
	chr5:116240088	F:CGGTTCTCTCCACTAACATTC<br>R:GTTAGAGGCATCAGGGATTTC	765
	chr5:151113455	F:CCCACCATCAACCTAGATTTG<br>R:GTCCTCACAGAACTCTCATTC	706
	chr1:62103725	F:GTGTGGACTGAGTTCTATAAGG<br>R:GATCACTTGAGGTCAGGAGTT	746
	chr1:146757199	F:GGCAGCACTTGGCATTTAACC<br>R:CTAGGCTCGGCTTGGATGTTC	760
	chr2:141699162	F:GCAGAGATCAGTTGCTTGTTC<br>R:CTGGGCTAGTGTTCTTAATGG	638

Primers used for validation of the off-target site from whole-genome analysis.

sgRNA-*Prrt2*-C	chr4:146185910	F:AAGGATACAAGGATCTCAGG<br>R:ACCACTCCGAAGATAATAGC	999
