# Supplementary material for: One-step generation of complete gene knockout mice and monkeys by CRISPR/Cas9-mediated gene editing with multiple sgRNAs
Source: Cell Res. 2017 Jun 6;27(7):933–45. doi: 10.1038/cr.2017.81 (PMC5518993; doi:10.1038/cr.2017.81)
Supplement: Supplementary information, Data S1 — C-CRISPR-mediated complete gene knockout in mice and monkeys. [file cr201781x8.pdf]

Supplementary information, Data S1

C-CRISPR-mediated complete gene knockout in mice and monkeys.

Gene deletion in mice

1. Choose the key exon of the gene for targeting.

2. Within DNA sequence of the key exon, design 4 to 6 adjacent sgRNAs (spaced 10-200 bps) with minimal off-target effects according to online software (http://crispr.mit.edu/).

Note: For genes located in only one allele, such as genes in Y chromosome, 2 to 3 adjacent sgRNAs are required. For genes located in autosomes, 4 to 6 adjacent sgRNAs are required.

Note: The majority of sgRNAs for gene editing in mice are very efficient. Therefore, we recommend using these sgRNAs directly for gene editing without pretest.

3. Synthetize DNA oligos for each sgRNA according to sgRNA sequence. Primers for PCR amplification are listed in the table below.

**Name**	**Sequence (5' to 3')**
sgRNA F	TAATACGACTCACTATAGGG-sgRNA (20 bp)-GTTTTAGAGCTAGAAATAG
sgRNA R	AAAAGCACCGACTCGGTGCC
Cas9 F	TAATACGACTCACTATAGGGAGATTTCAGGTTGGACCGGTG
Cas9 R	GACGTCAGCGTTCGAATTGC

4. Make the template for *in vitro* transcription of sgRNA:

A. Set up the following reaction mixture to amplify the DNA fragment from px330 for the sgRNA scaffold.

**Component**	**Volume**
10 × PCR Buffer for KOD -Plus- Neo	5μl
2 mM dNTPs	5μl
sgRNA F: (10μM)	2μl
sgRNA R: (10μM)	2μl
KOD -Plus- Neo (1 U/μl)	1μl
px330 (100ng/μl)	0.5μl
25 mM $MgSO_4$	3μl
ddH_2O	31.5μl
Total	50μl

B. Perform PCR using the following cycling conditions:

Cycle number	Denature	Anneal	Extend
1	95 ℃, 5min		
2-34	95 ℃, 30s	60 ℃, 30s	72 ℃, 30s
35			72 ℃, 5min

5. Purify T7-sgRNA PCR products, and then use them as the templates for IVT using

the MEGAshortscript T7 kit according to the kit protocol.

6. Purify the sgRNA individually using the MEGAclear kit and elute the RNA in 100 µl of TB buffer. Determine the RNA concentration by the Thermo Scientific NanoDrop 2000 spectrophotometer, and store it at -80 ℃.

7. Purchase or synthetize *Cas9* mRNA as following:

A. Set up the following reaction mixture to amplify the DNA fragment from px260 for *Cas9*.

**Component**	**Volume**
10×PCR Buffer for KOD -Plus- Neo	5µl
2 mM dNTPs	5µl
Cas9 F: (10µM)	2µl
Cas9 R: (10µM)	2µl
KOD -Plus- Neo (1 U/µl)	1µl
px260 (100ng/µl)	0.5µl
25 mM $MgSO_4$	3µl
ddH_2O	31.5µl
Total	50µl

B. Perform PCR using the following cycling conditions:

Cycle number	Denature	Extend
1	95 ℃, 5min	
2-34	95 ℃, 30s	68 ℃, 5min
35		72 ℃, 15min

8. Purify *T7-Cas9* PCR products and then use them as the templates for IVT using the mMESSAGE Mmachine T7 kit according to the kit protocol.

9. Purify the mRNA using the MEGAclear kit and elute the RNA in 100 µl of TB buffer. Determine the RNA concentration by the Thermo Scientific NanoDrop 2000 spectrophotometer, and store it at -80 ℃.
   Note: *Cas9* mRNA quality is the key for efficient DNA cleavage. Avoid *Cas9* mRNA degeneration.

10. *Cas9* mRNA (50 ng/µl) and sgRNAs (20-50 ng/µl for each sgRNA) are mixed and injected into the cytoplasm of fertilized eggs.
    Note: The total amount of sgRNAs should not exceed 200 ng/µl.

11. The injected zygotes are transferred into oviducts of pseudopregnant ICR females at 0.5 dpc. The embryos at different stages or mice could be directly used for phenotypic analysis.
    Note: Collect samples of embryos or mice for genotyping analysis to ensure complete gene deletion. Usually, more than 80% gene-edited embryos exhibited

large-fragment exon deletion at the target locus. Otherwise, design new sgRNAs to replace the sgRNAs with low DNA cleavage activity.

Gene deletion in monkey

1. Choose the key exon of the gene for targeting.

2. Within DNA sequence of the key exon, design ~ 20 sgRNAs with minimal off-target effects according to online software (http://www.rgenome.net/).

3. Synthetize sgRNAs individually as above method.

4. *Cas9* mRNA (100 ng/µl) and individual sgRNA (50 ng/µl for each sgRNA) are injected into monkey zygotes.

5. The injected zygotes are cultured to 8-cell stage and used for genotyping analysis. Four sgRNAs with highest DNA cleavage efficiency was selected for following gene editing.

6. *Cas9* mRNA (100 ng/µl) and sgRNAs mix (50 ng/µl for each sgRNA) are injected into monkey zygotes. And the embryos are transferred to surrogates.
